# Root-fungal interactions upon resource foraging in heterogeneous soils

**DOI:** 10.3389/fpls.2026.1899078

**Published:** 2026-08-31

**Authors:** Pavlína Stiblíková, Jan Jansa, Martin Rozmoš, Michala Kotianová, Marek Brindzák, Jan Šašek, Martin Weiser

**Affiliations:** 1 Department of Botany, Faculty of Science, Charles University, Prague, Czechia; 2 Laboratory of Fungal Biology, Institute of Microbiology of the Czech Academy of Sciences, Prague, Czechia

**Keywords:** mycorrhizal symbiosis, nutrient availability, root foraging precision, root–fungal cooperation, soil heterogeneity, herbs, quantitative real-time PCR (qPCR)

## Abstract

**Introduction:**

Plants respond to soil resource heterogeneity by foraging with their roots. The root foraging precision of plant species varies; and some species (especially those with low precision) may rely on symbiosis with mycorrhizal fungi to access nutrient-rich patches, thereby improving nutrient acquisition. However, the way herbaceous plants and arbuscular mycorrhizal fungi (AMF) interact during nutrient foraging is poorly understood.

**Methods:**

We tested nine mycorrhizal herbaceous plant species in a greenhouse experiment by exposing them to soil with heterogeneous or homogeneous nutrient distribution and with or without AMF inoculation. Root-foraging precision, shoot and root biomass production, and root-associated AMF gene copy number were quantified using plant biomass measurements and qPCR analyses of AMF.

**Results:**

Root foraging precision (in terms of roots placement) differed between species. Although foraging was evident in the heterogeneous nutrient treatment, it was largely unaffected by mycorrhizal inoculation. The exception was Melilotus officinalis which showed reduced root foraging precision with increasing root-associated AMF gene copy number. Plant biomass responses to AMF were species-specific, with overall root and shoot biomass declining slightly with increasing root-associated AMF gene copy number.

**Discussion:**

These results suggest that AMF inoculation generally does not alter root foraging precision across species, supporting the view that foraging precision is a species-specific trait independent of the collaboration gradient in the root economic spectrum. The balance between root foraging and mycorrhizal symbiosis may depend on other factors such as nutrient stoichiometry and forms. Future studies should thus examine root foraging, extraradical hyphal development and nutrient transfer under varying levels of nutrient limitation and spatial heterogeneity to better understand plants’ nutrient acquisition strategies.

## 1 Introduction

To acquire a sufficient amount of nutrients, plants often form various associations with other organisms, amongst the most important being mycorrhizal symbiosis. Over 90% of vascular plants establish some type of mycorrhizal association, most often with arbuscular mycorrhizal fungi (AMF) (Brundrett and Tedersoo, 2018). Plant roots and mycorrhizal hyphae cooperate to different degrees to acquire nutrients, forming a continuum of collaboration strategies that range from ‘do-it-yourself’ to ‘outsourcing’ approaches to nutrient uptake (Brundrett, 2009; Bergmann et al., 2020). The degree of cooperation depends on the characteristics of the two partners, as well as on environmental conditions (Johnson, 2010; Unger et al., 2016; Zhang et al., 2019; Šmilauer et al., 2020). In heterogeneous soil environments, plants may forage for nutrients using either their roots or mycorrhizal hyphae (Drew, 1975; John et al., 1983; Cavagnaro et al., 2005; Shi et al., 2011). Foraging precision appears to be a species-specific trait of both plants and fungi (Campbell et al., 1991; Cavagnaro et al., 2005; Kembel and Cahill, 2005; Weiser et al., 2016; Šmilauer et al., 2020) and may, therefore, be distributed along the collaboration continuum. This has been demonstrated for trees (Cheng et al., 2016) but remains still poorly understood for herbaceous species. Furthermore, despite the importance of mycorrhizal fungi for plant nutrient acquisition, root foraging experiments with herbaceous species are often conducted without controlling for mycorrhizal colonisation of plants and frequently in the absence of mycorrhiza altogether.

In a heterogeneous soil environment, plants’ association with AMF may help compensate for limited root foraging abilities (Cui and Caldwell, 1996b). Compared to roots, hyphae of AMF rapidly colonise soil, may better compete with other microorganisms for nutrients from nutrient-rich patches and can exploit smaller pores (Friese and Allen, 1991; Allen, 2007). The cost of producing new hyphae is lower than that of producing new roots (Tibbett, 2000; Hodge, 2006). The balance between the do-it-yourself strategy and outsourcing of nutrient acquisition to AMF (Bergmann et al., 2020) supports the existence of a root–hyphae foraging trade-off (but see Stiblíková et al., 2023). Indeed, a trade-off in foraging strategies between trees and AMF or ectomycorrhizal fungi has been reported (e.g. Eissenstat et al., 2015; Liu et al., 2015; Chen et al., 2016; Cheng et al., 2016). AMF-associated tree species tend to be more precise root foragers, whereas ectomycorrhizal fungi-associated tree species are less precise foragers and rely more heavily on fungal-mediated nutrient acquisition. However, this trade-off is poorly understood for herbaceous species.

Only a few studies have directly examined root foraging precision of herbaceous plants under the influence of mycorrhizal associations, with results showing either no change in root foraging after the addition of AMF (Farley and Fitter, 1999b; Hodge, 2001; Wijesinghe et al., 2001) or a reduction in root foraging precision in their presence (Cui and Caldwell, 1996a; Felderer et al., 2013). These contrasting results may reflect differences in mycorrhizal colonisation rates, which were often quite low or measured only as the presence of arbuscules in roots (Farley and Fitter, 1999b; Wijesinghe et al., 2001). Molecular quantification of AMF in roots and/or soil substrate, which is commonly available today, provides an additional approach for characterising plant–AMF associations (Felderer et al., 2013). Successful inoculation does not necessarily result in effective mycorrhizal colonization, as its establishment depends on multiple factors such as host-fungus compatibility and environmental conditions (e.g. nutrient and water availability [Mason et al., 2000; Klironomos, 2003; Lekberg and Koide, 2008; Frew, 2023]). Therefore, quantifying associated AMF gene copies using quantitative real-time PCR (qPCR) provides a more informative approach for evaluating mycorrhizal effects, allowing them to be analysed not only as a categorical factor (inoculated vs. non-inoculated) but also in relation to the actual amount of fungal DNA present in plant roots. Differences in the nature of nutrient patches used (organic, single-nutrient, or complex-nutrient patches) also complicate comparisons between experiments. In our previous study on root foraging and mycorrhizal symbiosis, no clear pattern of a trade-off along the collaboration gradient was observed, although the data were compiled from multiple databases rather than from a single experiment (Stiblíková et al., 2023). From the available evidence, the degree of plant cooperation with mycorrhizal fungi during root foraging for nutrients appears to be species-specific; however, additional evidence is needed to confirm this.

To further investigate root–fungal interactions during root foraging for heterogeneous nutrient sources, the present study linked the root allocation patterns of nine grassland species in heterogeneous nutrient environments with the presence of AMF in their roots. We examined how plant root foraging ability and biomass were affected by both the presence and the number of root-associated AMF gene copies in the central part of the root system. Plant species were grown in pots with either heterogeneous or homogeneous nutrient distributions and with or without mycorrhizal inoculum. Root dry biomass served as a proxy for plant foraging precision, and root-associated AMF nuclear large ribosomal subunit (LSU) copy numbers in the central part of the root system were quantified using qPCR. Our null hypothesis was that plant root foraging precision was unaffected by the presence of root-associated AMF LSU copy number. Our alternative hypothesis was that root foraging precision would be affected by the presence and number of root-associated AMF LSU copies, with higher root-associated AMF LSU copy number being associated with lower root foraging precision. Because we did not measure the distribution of extraradical hyphae, nutrient uptake by fungal hyphae, or nutrient transfer to plants from hyphae (e.g., using isotopes), our analyses were limited to testing associations between root foraging precision and root-associated AMF gene copies rather than the mechanism underlying root and hyphal foraging.

## 2 Materials and methods

### 2.1 Plant species selection, substrate, and mycorrhizal inoculum

We selected nine herbaceous plant species occurring in meadow habitats in Czechia: *Centaurea jacea* L. (Asteraceae), *Lotus corniculatus* L. and *Melilotus officinalis* (L.) Lam. (Fabaceae), *Lythrum salicaria* L. (Lythraceae), *Linaria vulgaris* Mill. (Plantaginaceae), *Anthoxanthum odoratum* L. and *Phleum phleoides* (L.) H.Karst. (Poaceae), *Ranunculus acris* L. (Ranunculaceae), and *Potentilla recta* L. (Rosaceae). All selected species form symbiosis with AMF. Their ability to establish mycorrhizal symbiosis with the inoculum used in this study was tested beforehand in a pre-experiment including 24 species (see **Supplementary Table 1** in the **Supporting Information**). We considered the relative representation of different plant families according to their presence in the flora of Czechia and the different rates of root foraging precision established in our previous experiments (Weiser et al., 2016). We obtained plant seeds from a commercial supplier (Planta Naturalis Ltd., Markvartice, Czechia, www.plantanaturalis.com).

For the substrate in this experiment, we used a mixture of autoclaved (at 100–120 °C/6 h) zeolite, autoclaved sand, gamma-sterilised soil (min. 25 kGy), and mycorrhizal/non-mycorrhizal inoculum at the proportions of 43%, 43%, 9%, and 5%, respectively. The mycorrhizal inoculum consisted of spores of three fungal species – *Funneliformis mosseae* (T.H.Nicolson & Gerd.) C.Walker & A. Schuessler, *Entrophospora claroidea* (N.C.Schenck & G.S.Sm.) Błaszk., Niezgoda, B.T.Goto & Magurno, and *Rhizophagus irregularis* (Błaszk., Wubet, Renker & Buscot) C.Walker & A.Schüsler – produced separately, and mixed together with chopped fragments of leek roots (*Allium porrum* L.; less than 2 cm in length) colonised by these fungal species, and substrate from the leek rhizosphere containing extraradical mycelial fragments of the respective fungi. The non-mycorrhizal inoculum contained leek root fragments and substrate prepared in the same way but without AMF propagules.

### 2.2 Experiment

In the experiment, we applied two types of treatment – mycorrhizal or non-mycorrhizal inoculum and heterogeneous or homogeneous nutrient distribution – in a full-factorial design. Each of the four treatment combinations was replicated 10 times for each species, resulting in a total of 360 pots. For the nutrient treatments, we used a drip irrigator with flow-powered proportional dosing pumps (Dosatron, D25RE2) that added fertiliser to the water. Fertilised water was then delivered by the drippers to the pots at a constant amount at the same time. Each pot was supplied with two drippers placed at the opposite sides of the pot approximately 1 cm from the edge (**Supplementary Figure 1B** in the **Supporting Information**). In the heterogeneous treatment, a dripper supplied water with fertiliser to one side of the pot, whilst a second dripper supplied water without fertiliser to the other side. In the homogeneous treatment, each pot received water with fertiliser from both drippers. To create heterogeneous and homogeneous nutrient treatments, we changed the proportion of the fertiliser in the drippers. We used the recommended fertiliser concentrations for adult plants (0.1% v/v solution; Wuxal Super, NPK 8:8:6 + micronutrients, Aglukon; **Supplementary Table 2** in the **Supporting Information**). The fertiliser was mixed with water (10:1) in storage barrels and then diluted further to obtain a 2% solution for the heterogeneous treatment and a 1% solution for the homogeneous treatment. Thus, both treatments received the same total amount of fertiliser. We placed the pots on water-levelled perforated tables to allow excess water to drain freely. We irrigated the pots twice a day for 10 minutes. The method used to create nutrient treatments is a modified version of Campbell and Grime (1989) nutrient-dripping method. The non-retentive substrate in the pots ensured that the main flow of the water with fertiliser was vertical, thus creating heterogeneity with only limited mixing in a horizontal direction without using a physical barrier.

Before the experiment, we sterilised the seeds with a solution of sodium hypochlorite (5% w/w) and water at a ratio of 1:10 for 10 minutes and rinsed them with demineralised water. We also sterilised the equipment and pots by washing them with 60% ethyl alcohol and wiping them with paper towels. On 22 April 2019, we sowed the seeds in Petri dishes containing white silica sand sterilised in an autoclave (120 °C/6 h). We placed the Petri dishes in a growth chamber (16:8 h day/night cycle with 20/15 °C temperature, respectively). After three weeks, we transplanted seedlings into small square pots (7 × 7 × 8 cm, 0.23 L) filled with the prepared substrate (sand, zeolite, soil, and mycorrhizal/non-mycorrhizal inoculum) and a nonwoven fabric at the bottom to prevent roots from growing out of the pot. Each of these small pots was perforated on two opposite sides with multiple 7-mm holes and placed inside a similar pot without perforations to keep the substrate in the dark. The perforated area was at least 60% of the pot wall surface (**Supplementary Figure 1A** in the **Supporting Information**). For each species, 40 small pots were used, half with mycorrhizal and half with non-mycorrhizal inoculum (in total 360 small pots). Seedlings grew in the small pots for six weeks in the greenhouse and were watered regularly with tap water. During this time, plants had enough time to establish symbiosis with mycorrhizal fungi.

On June 24, we transplanted the plants into round 3-L pots (TEKU Pöppelman, MCI 19) filled with the same substrate as the small pots. We removed each small, perforated pot from the non-perforated pot and placed it, with the plant, in the middle of a 3-L pot (**Supplementary Figure 1B** in the **Supporting Information**). We installed drip irrigation in the large pot and ensured that the perforated sides of the small pot faced the drippers. Roots and fungal hyphae could grow freely through the perforations of the small pot into the substrate in the large pot. We started the nutrient treatments as described above and let the plants grow for five weeks.

We harvested the plants after five weeks on 29 July. First, we removed the aboveground parts of the plant; we then cut out the small pot with a sharp knife (we cut off the roots that grew through the holes) and carefully removed it from the large pot. We harvested all the roots from the small pot by washing off the substrate. Then, we divided the rest of the substrate in the large pot by cutting it with a sharpened metal plate. The plane of the cut was vertical and perpendicular to the line connecting the positions of the two drippers, thus perpendicular to the nutrient gradient in the pots with the heterogeneous treatment. We thus obtained the nutrient-rich and nutrient-poor parts of the substrate, together with roots, from the heterogeneous treatment pots (or two similar parts from the homogeneous treatment pots). We harvested all roots from each half of the pot by washing off the substrate. Shoot and root biomass was dried at 65 °C for 48 hours and then weighed. The roots from the small central pots were subsequently analysed using qPCR. The experiments were performed in the greenhouse of the experimental garden of the Faculty of Science, Charles University (50.069N, 14.425E). The greenhouse was not equipped with automatic air conditioning; thus, temperature regulation was limited to ventilation, water misting, and shading with shade cloth. However, neither soil nor air temperature was measured during the experiment.

### 2.3 qPCR analyses

For each plant species, we performed qPCR analyses of dried root biomass collected from the small central pots (10 mycorrhizal and 10 non-mycorrhizal pots in either the heterogeneous or the homogeneous nutrient treatment; 40 pots per species). Dried roots were milled in a ball mill (MM 200, Retsch, Haan, Germany) to physically homogenise the root samples and increase the representativeness of subsamples. Then, we extracted DNA from 10–12.5 mg of milled root powder using the glassmilk method described by Gryndler et al. (2014). To assess DNA extraction efficiency, we added 1.8 × 10^10^ gene copies of an internal standard (a linearised plasmid carrying a fragment of cassava mosaic virus, GenBank accession AJ427910) to each sample before DNA extraction (Thonar et al., 2012). qPCR analyses were performed using Luna^®^ Universal Probe qPCR Master Mix (New England Biolabs) on a Lightcycler 480 instrument (Roche). We followed the protocol described by Thonar et al. (2012), with species-specific markers for *F. mosseae* (marker ‘moss’), *R. irregularis* (marker ‘intra’), and *E. claroidea* (marker ‘clar’). All markers targeted the nuclear large ribosomal subunit (LSU) of the respective AMF taxa. We converted the qPCR results (Cq values) to root-associated LSU copy numbers of individual AMF taxa per unit weight (=mg) of roots, and we corrected them for extraction efficiency based on the recovery of the internal standard according to Thonar et al. (2012). One sample (belonging to the species *L. salicaria*, highlighted by red colour in **Supplementary Table 3** in the **Supporting Information**) was excluded from the data analyses due to very low DNA recovery, most likely resulting from the small amount of available root material, which rendered the corrected qPCR estimate of mycorrhizal abundance unreliable.

### 2.4 Data preparation and analysis

**Figure 1:**
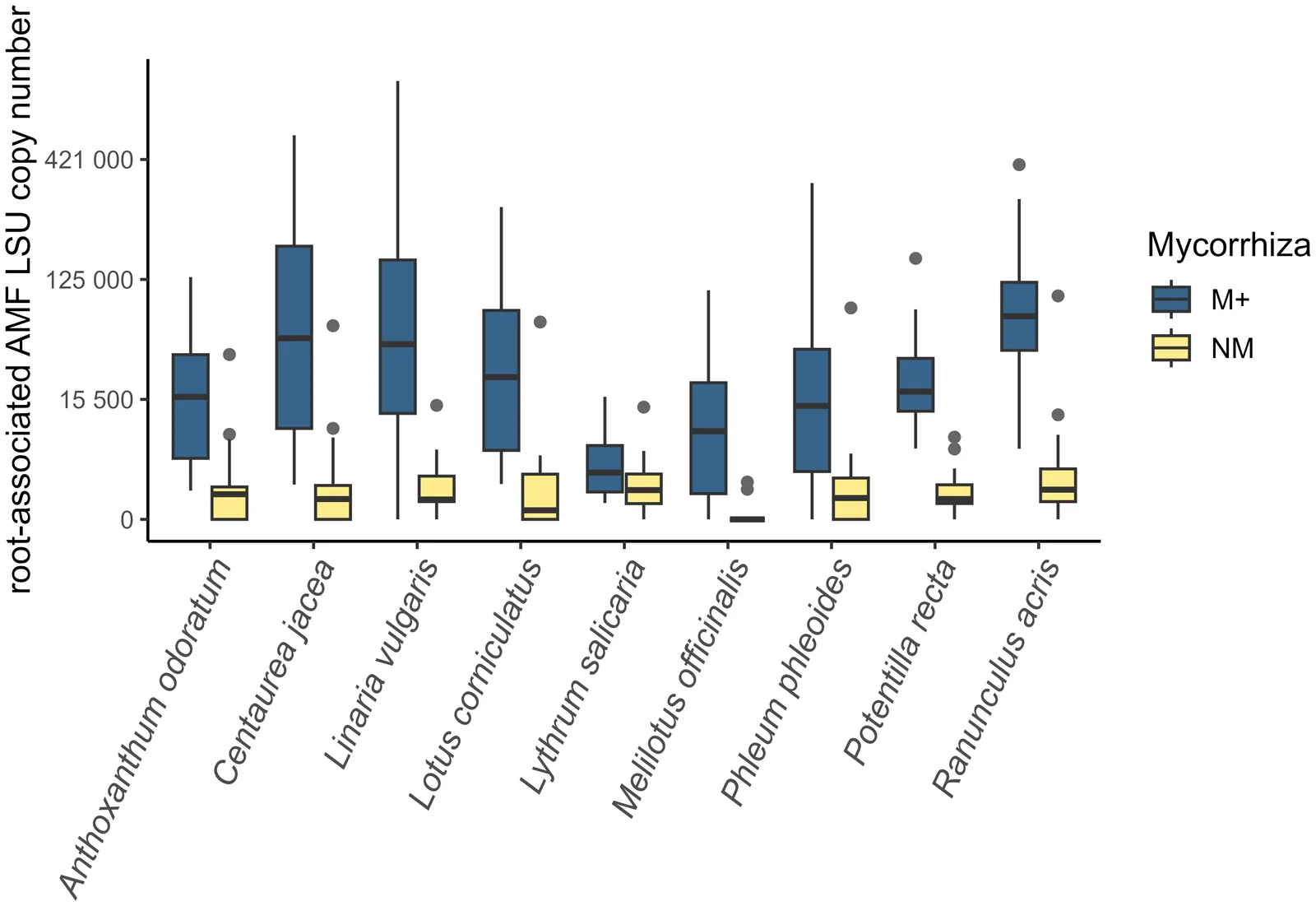
The root-associated AMF LSU copy number of the three inoculated mycorrhizal fungal taxa per unit weight of roots in mycorrhizal (dark blue boxplots) and non-mycorrhizal (yellow boxplots) treatments for nine plant species. The non-mycorrhizal treatment contained no or very low numbers of root-associated AMF LSU copy number (caused by contamination of pots with mycorrhizal fungi or by false-positive signals from qPCR).

In the analyses, the aboveground biomass was denoted ‘shoot biomass’. ‘Root system biomass’ referred to the biomass of roots from both halves of the large pot together with roots from the small central pot. ‘Whole plant biomass’ was the shoot and root system biomass combined. ‘Root:shoot (R:S) ratio’ was calculated from the shoot and root system biomass. All biomass data were log-transformed in all subsequent analyses to correct for non-normality. For calculation of root foraging precision, we used only root biomass from the separated halves of the substrate from the large pot. We calculated root foraging precision in each pot as the decadic logarithm of N/W, where N was the biomass of dried roots from the nutrient-rich half of a given pot and W was the biomass of dried roots from the nutrient-poor half of the same pot. Values above zero indicated that plants allocated more roots to the nutrient-rich half of the pot, whereas values below zero indicated that plants allocated more roots to the nutrient-poor half of the pot. In the case of homogeneous pots, we assigned N or W randomly to one of the two halves. Thirty-seven plants died or were lost during sampling, three samples contained missing qPCR values, and one sample was excluded after qPCR analysis due to very low DNA recovery; thus, we used 319 pots from the original 360 pots in the analyses (**Supplementary Table 4** in the **Supporting Information**). ‘Root-associated AMF LSU copy number’ in small central pots was the gene copy number of LSU of individual AMF taxa (*F. mosseae*, *E. claroidea*, and *R. irregularis)* per unit weight of roots summed together. In the case of fungal presence, 98% of AMF-inoculated pots showed a positive presence of *F. mosseae*, 51% showed the presence of *R. irregularis*, and 23% showed the presence of *E. claroidea* (**Supplementary Tables 3, 7** in the **Supporting Information**). As *E. claroidea* was not detected in 77% of the AMF-inoculated pots, we decided to analyse all three AMF taxa together. Although AMF contamination (or false positive detection) was encountered in some non-inoculated control pots (numbers were not zero), root-associated AMF LSU copy number was lower than in most successfully colonised inoculated pots (**Supplementary Table 3** in the **Supporting Information**, **Figure 1**). For the variable ‘root-associated AMF LSU copy number’, we applied a cube-root transformation in all subsequent analyses to correct for non-normality of the data. This transformation reduced the values from millions of root-associated AMF LSU copy numbers to thousands in units on the transformed scale. We chose cube-root transformation because it effectively decreases right skewness and can also be applied to zeros, which were frequent in our dataset.

**Figure 2:**
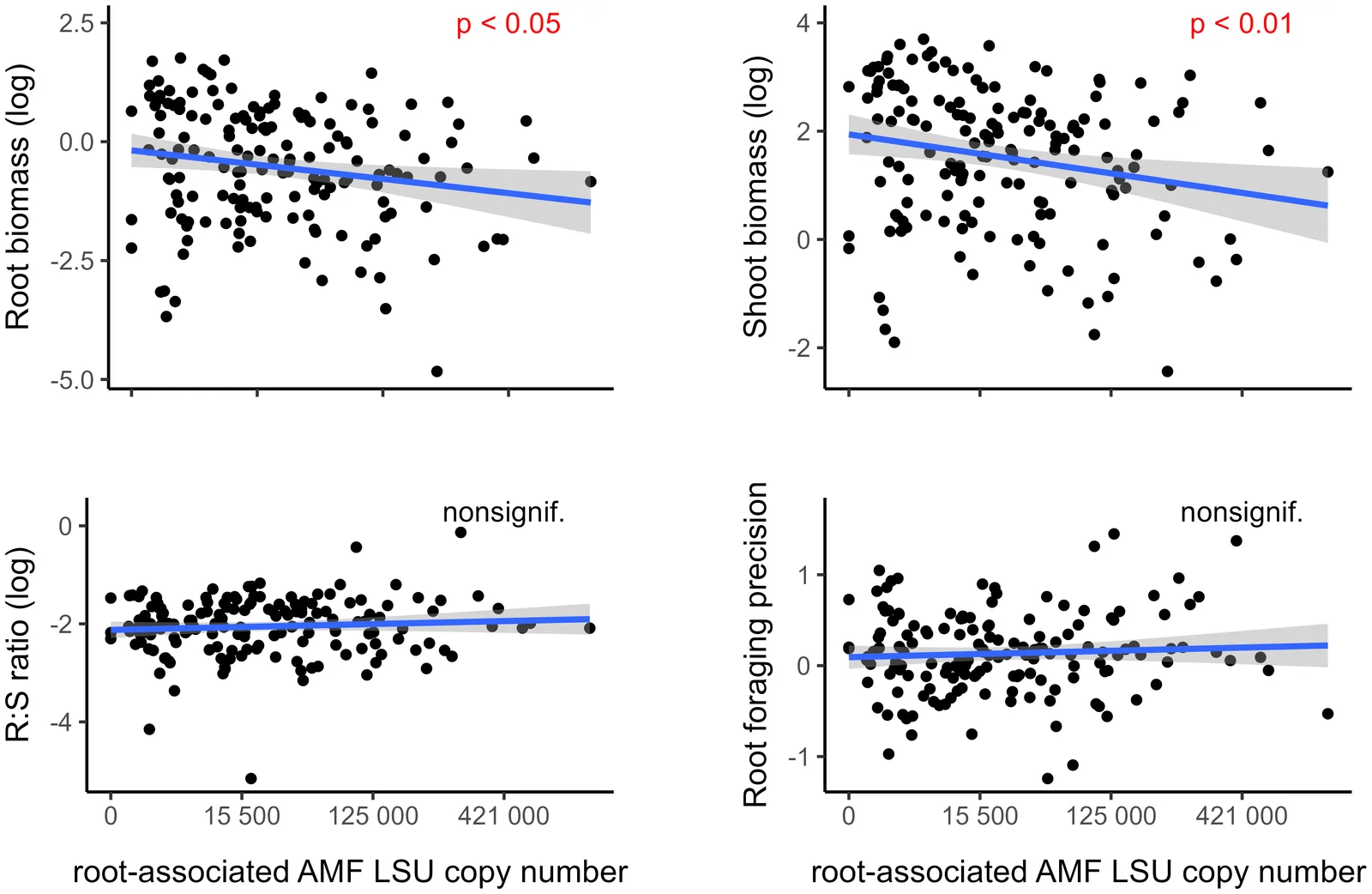
The effects of the root-associated AMF LSU copy number per unit weight of roots of the three mycorrhizal fungal inoculants on root and shoot biomass, root:shoot (R:S) ratio and root foraging precision of nine plant species. All biomass data were log-transformed. We analysed only data from pots inoculated with the living mycorrhizal fungi. We found a significant decline in root and shoot biomass with increasing root-associated AMF LSU copy number. Root foraging precision values above zero indicate that plants allocated more roots to the nutrient-rich half of the pot, and values below zero indicate that plants allocated more roots to the nutrient-poor half of the pot. Each point represents data from one pot, and the blue line is the regression line with confidence intervals.

To test whether the inoculation was successful, we used a linear model to explore the effect of mycorrhizal treatment (mycorrhizal or non-mycorrhizal inoculation), in combination with species identity and nutrient treatment, on root-associated AMF LSU copy number of each pot. Then, we estimated the effects of mycorrhizal treatment (mycorrhizal/non-mycorrhizal pots or the root-associated AMF LSU copy number), species identity, and nutrient treatment (both individually and in combination) on plant biomass (root system biomass, shoot biomass, whole plant biomass, and R:S ratio) and root foraging precision using linear models. Furthermore, we explored root and shoot biomass, root foraging precision and mycorrhizal colonisation at the level of individual species. We determined partial correlations of root and shoot biomass with mycorrhizal treatment as a category and partial correlations of root foraging precision with root-associated AMF LSU copy number for individual species using Welch’s two-sample *t*-test. We used data from mycorrhizal and non-mycorrhizal pots for analyses of root-associated AMF LSU copy number even though there was quite a high number of zeros. The results of analysis of data only with the mycorrhizal treatment were almost the same (**Figure 2**, **Supplementary Table 5** in the **Supporting Information**). We fit all models using R (R Core Team, 2024, version 4.4.0) in Rstudio (Posit Team, 2024, version 2024.4.1.748). All images were created using the ggplot2 package (Wickham, 2016) and the cowplot package (Wilke et al., 2024).

## 3 Results

For the analyses that included plant biomass as the response variable, we show results only for root biomass and shoot biomass as they were highly correlated with whole-plant biomass (**Supplementary Table 6** in the Supporting Information). The results of all the analyses were similar.

### 3.1 Success of mycorrhizal inoculation

The mycorrhizal pots showed significantly higher root-associated AMF LSU copy number compared to non-mycorrhizal pots, although the numbers were highly variable in the mycorrhizal treatment and the level of difference between mycorrhizal and non-mycorrhizal pots depended also on the plant species (**Figure 1**, **Table 1**, **Supplementary Table 3** in the **Supporting Information**). Root-associated AMF LSU copy number also differed between plant species irrespectively of the mycorrhizal inoculation treatment. The mycorrhizal inoculation was measured only using qPCR analyses; we did not measure microscopical colonization data, extraradical hyphal density or plant tissue nutrient concentration to evaluate the success of mycorrhizal inoculation.

**Table 1:** The effects of mycorrhizal inoculation (Mycorrhiza: inoculated vs. non-inoculated), plant species identity (Species) and nutrient treatment (Nutrient: homogeneous vs. heterogeneous) on the root-associated AMF LSU copy number of the three mycorrhizal fungal inoculants per unit weight of roots.

Source of variation (factor/factor interaction)	Df	Sum Sq	Mean Sq	F value	Pr (>F)
**Mycorrhiza**	1	59901	59901	234.723	**<0.001**
**Species**	8	12782	1598	6.261	**<0.001**
Nutrient	1	86	86	0.338	0.561
**Mycorrhiza × Species**	8	8846	1106	4.333	**<0.001**
Mycorrhiza **× Nutrient**	1	581	581	2.276	0.133
**Species × Nutrient**	8	3875	484	1.898	0.06
Mycorrhiza **× Species × Nutrient**	8	1788	223	0.876	0.537
Residuals	283	72222	255		

Significant results are in bold. Root-associated AMF LSU copy number was cube-root transformed for the statistical analysis.

### 3.2 Biomass and root foraging in different treatments

**Figure 3:**
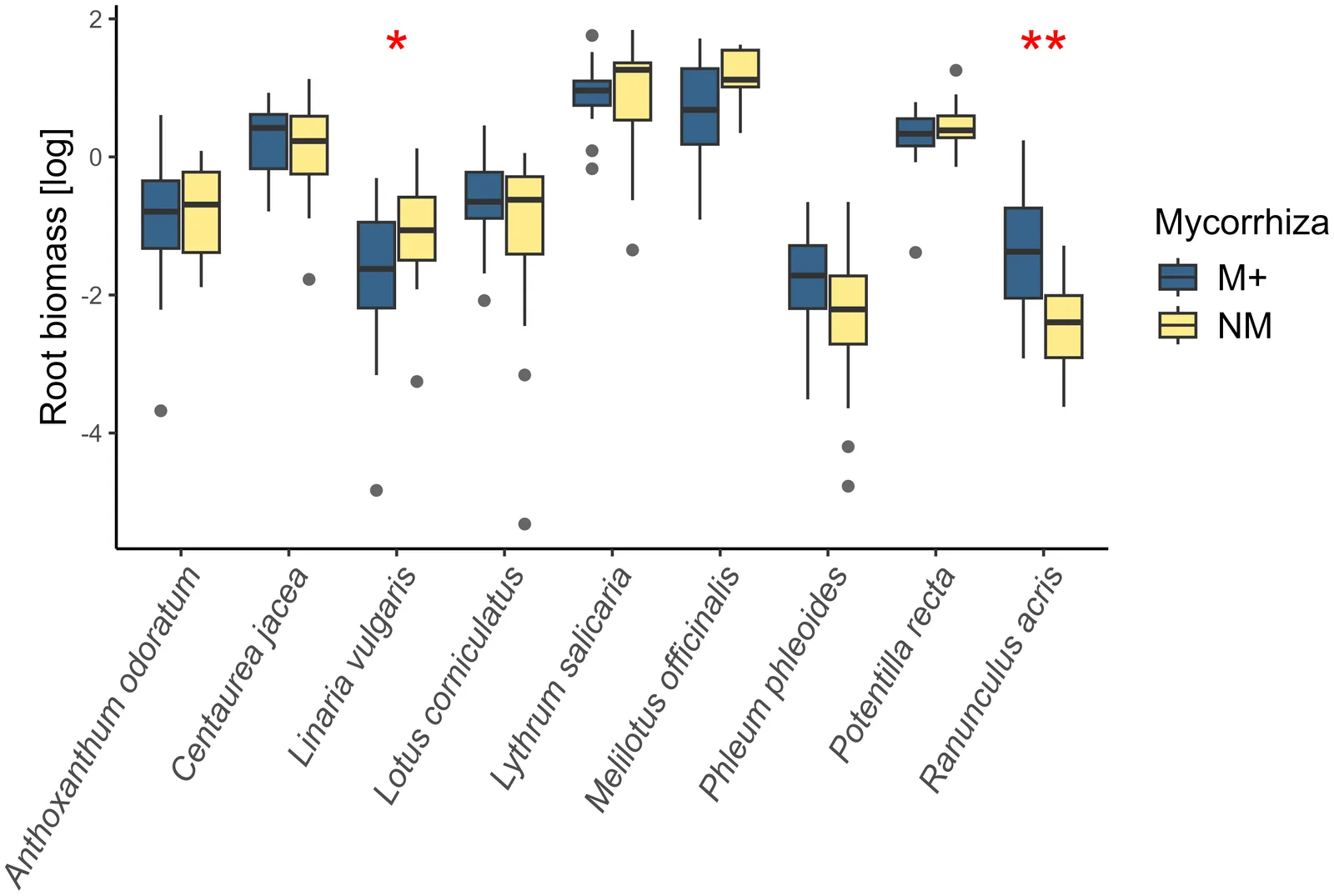
The effects of the presence of mycorrhizal fungi on root biomass of nine plant species. The dark blue boxplots represent root biomass in pots with mycorrhizal inoculum (M+), the yellow boxplots represent root biomass in pots without mycorrhizal inoculum (NM). Root biomass was log-transformed. Significant differences between mycorrhizal and non-mycorrhizal treatments are marked with asterisks for each species.

Species identity, nutrient treatment, and mycorrhizal treatment (presence/absence of AMF) in interaction with species identity significantly affected root (and shoot) biomass of some plant species (**Table 2**, **Figure 3**). The root biomass and shoot biomass of *R. acris* were significantly higher in the mycorrhizal treatment (root: *t* = 3.01, p < 0.01; shoot: *t* = 2.26, p < 0.05) compared to the non-mycorrhizal treatment. In contrast, root biomass, but not shoot biomass, of *L. vulgaris* was significantly lower in the mycorrhizal treatment (*t* = -1.95, p < 0.05). Root biomass was slightly higher in the heterogeneous nutrient treatment, but not significantly so, compared to the homogeneous nutrient treatment in the test of nutrient treatments only (*t* = -1.00, p > 0.05). R:S ratio differed significantly only between species; mycorrhizal treatment and nutrient treatment and any of the possible interactions had no effect on R:S ratio (**Table 2**).

**Table 2:** The effects of mycorrhizal inoculation (Mycorrhiza: inoculated vs. non-inoculated), plant species identity (Species), nutrient treatment (Nutrient: homogeneous vs. heterogeneous) and their interactions on root and shoot biomass, root:shoot (R:S) ratio and root foraging precision of nine plant species.

Response variable	Source of variation (factor/factor interaction)	Df	Sum Sq	Mean Sq	F value	Pr (>F)
Root biomass	Mycorrhiza	1	0.01	0.04	0.057	0.811
	**Species**	8	371.8	46.48	73.812	**<0.001**
	**Nutrient**	1	2.8	2.82	4.473	**0.035**
	**Mycorrhiza × Species**	8	18	2.24	3.564	**<0.001**
	Mycorrhiza × Nutrient	1	1	1.04	1.654	0.199
	Species × Nutrient	8	0.6	0.07	0.117	0.999
	Mycorrhiza × Species × Nutrient	8	6.1	0.77	1.22	0.287
	Residuals	282	177.6	0.63		
Shoot biomass	Mycorrhiza	1	0	0.03	0.048	0.827
	**Species**	8	408.1	51.01	86.545	**<0.001**
	Nutrient	1	1.7	1.67	2.837	0.093
	**Mycorrhiza × Species**	8	13.8	1.73	2.937	**0.004**
	Mycorrhiza × Nutrient	1	0.8	0.85	1.437	0.232
	Species × Nutrient	8	2.2	0.28	0.469	0.878
	Mycorrhiza × Species ×Nutrient	8	6.5	0.81	1.372	0.208
	Residuals	282	166.2	0.59		
R:S ratio	Mycorrhiza	1	0.13	0.125	0.764	0.383
	**Species**	8	53.99	6.749	41.19	**<0.001**
	Nutrient	1	0.01	0.006	0.039	0.845
	Mycorrhiza × Species	8	0.81	0.101	0.616	0.765
	Mycorrhiza × Nutrient	1	0.15	0.147	0.895	0.345
	Species × Nutrient	8	0.8	0.1	0.608	0.771
	Mycorrhiza × Species × Nutrient	8	1.51	0.189	1.153	0.328
	Residuals	281	46.04	0.164		
Root-foraging precision	Mycorrhiza	1	0.13	0.131	0.681	0.41
	Species	8	1.11	0.139	0.725	0.67
	**Nutrient**	1	7.25	7.251	37.802	**<0.001**
	Mycorrhiza × Species	8	1.67	0.208	1.087	0.373
	Mycorrhiza × Nutrient	1	0.01	0.006	0.031	0.861
	**Species × Nutrient**	8	3.44	0.43	2.243	**0.025**
	Mycorrhiza × Species × Nutrient	8	1.55	0.194	1.013	0.426
	Residuals	283	54.28	0.192		

Significant results are in bold. Root and shoot biomass and R:S ratio were log-transformed for the statistical analyses.

Root foraging precision differed between heterogeneous and homogeneous nutrient treatments (it was higher in the heterogeneous treatment) and between species (the nutrient treatment × species identity interaction was significant) (**Table 2**, **Supplementary Figure 2** in the **Supporting Information**). All species showed the same trend of higher root foraging precision in the heterogeneous nutrient treatment compared to the homogeneous treatment (significant results: *C. jacea*: *t* = -3.15, p < 0.01; *L. corniculatus*: *t* = -3.89, p < 0.001; *L. salicaria*: *t* = -2.45, p < 0.05; *M. officinalis*: *t* = -2.79, p < 0.05; *P*. *recta*: *t* = -6.18, p < 0.001). Mycorrhizal treatment had no effect on root foraging precision (**Table 2**).

However, some pots assigned to the mycorrhizal treatment were almost non-mycorrhizal (**Figure 1**, **Supplementary Table 3** in the **Supporting Information**). Thus, it was reasonable to test the presence and abundance of mycorrhizal fungi using root-associated AMF LSU copy number. Mycorrhizal treatment (root-associated AMF LSU copy number) significantly affected root and shoot biomass (**Table 3**, **Figure 2**). Both root biomass and shoot biomass of plants declined with increasing root-associated AMF LSU copy number (root biomass: F_1_, _316_ = 5.93, p < 0.05, *R²* = 0.018; shoot biomass: F_1_,_316_ = 6.76, p < 0.01, *R²* = 0.021). Results for R:S ratio were almost similar whether the root-associated AMF LSU copy number was used or whether AMF presence/absence was used in the analysis. However, the nutrient treatment × mycorrhiza interaction had a weak but significant effect when the root-associated AMF LSU copy number was used (**Table 3**). The R:S ratio of plants in the heterogeneous nutrient treatment was positively correlated with the root-associated AMF LSU copy number, whereas for plants in the homogeneous treatment, the trend was the opposite, although these effects were not significant when homogeneous and heterogeneous nutrient treatments were analysed separately. Moreover, the significant effect of the nutrient treatment × mycorrhiza interaction on the R:S ratio disappeared when we used only mycorrhizal inoculated pots for the analyses (**Supplementary Table 5** in the **Supporting Information**).

**Table 3:** Linear model analyses of the effects of mycorrhizal root colonisation (Mycorrhiza: root-associated AMF LSU copy number), plant species identity (Species) and nutrient treatment (Nutrient: homogeneous vs. heterogeneous) on the root and shoot biomass, root:shoot (R:S) ratio and root foraging precision of plants.

Response variable	Source of variation (factor/factor interaction)	Df	Sum Sq	Mean Sq	F value	Pr (>F)
Root biomass	**Mycorrhiza**	1	10.6	10.64	15.561	**<0.001**
	**Species**	8	360.9	45.11	65.948	**<0.001**
	**Nutrient**	1	2.8	2.81	4.107	**0.044**
	Mycorrhiza × Species	8	6.8	0.85	1.249	0.271
	Mycorrhiza × Nutrient	1	1.2	1.2	1.757	0.186
	Species × Nutrient	8	0.4	0.05	0.072	1.000
	Mycorrhiza × Species × Nutrient	8	2.3	0.29	0.421	0.908
	Residuals	282	192.9	0.68		
Shoot biomass	**Mycorrhiza**	1	12.6	12.56	19.704	**<0.001**
	**Species**	8	395	49.38	77.475	**<0.001**
	Nutrient	1	1.7	1.73	2.707	0.101
	Mycorrhiza × Species	8	5.4	0.67	1.054	0.396
	Mycorrhiza × Nutrient	1	0.3	0.27	0.429	0.513
	Species × Nutrient	8	1.8	0.23	0.355	0.943
	Mycorrhiza × Species × Nutrient	8	2.9	0.36	0.569	0.803
	Residuals	282	179.7	0.64		
R:S ratio	Mycorrhiza	1	0.01	0.005	0.033	0.856
	**Species**	8	54.09	6.762	42.62	**<0.001**
	Nutrient	1	0	0.004	0.026	0.873
	Mycorrhiza × Species	8	2.05	0.257	1.618	0.119
	**Mycorrhiza × Nutrient**	1	0.8	0.798	5.03	**0.026**
	Species × Nutrient	8	0.69	0.086	0.54	0.826
	Mycorrhiza × Species × Nutrient	8	1.2	0.151	0.949	0.476
	Residuals	281	44.58	0.159		
Root-foraging precision	Mycorrhiza	1	0.19	0.188	1.026	0.312
	Species	8	1.09	0.137	0.747	0.65
	**Nutrient**	1	7.21	7.212	39.351	**<0.001**
	**Mycorrhiza × Species**	8	4.34	0.542	2.959	**0.003**
	Mycorrhiza × Nutrient	1	0	0	0.002	0.967
	**Species × Nutrient**	8	3.47	0.434	2.368	**0.018**
	Mycorrhiza × Species × Nutrient	8	1.28	0.16	0.873	0.539
	Residuals	283	51.86	0.183		

Mycorrhizal colonisation of roots was expressed as the root-associated AMF LSU copy number of each of the three mycorrhizal inoculants per unit weight of roots. Significant results are in bold. Root and shoot biomass and R:S ratio were log-transformed. The root-associated AMF LSU copy number was cube-root transformed.

**Figure 4:**
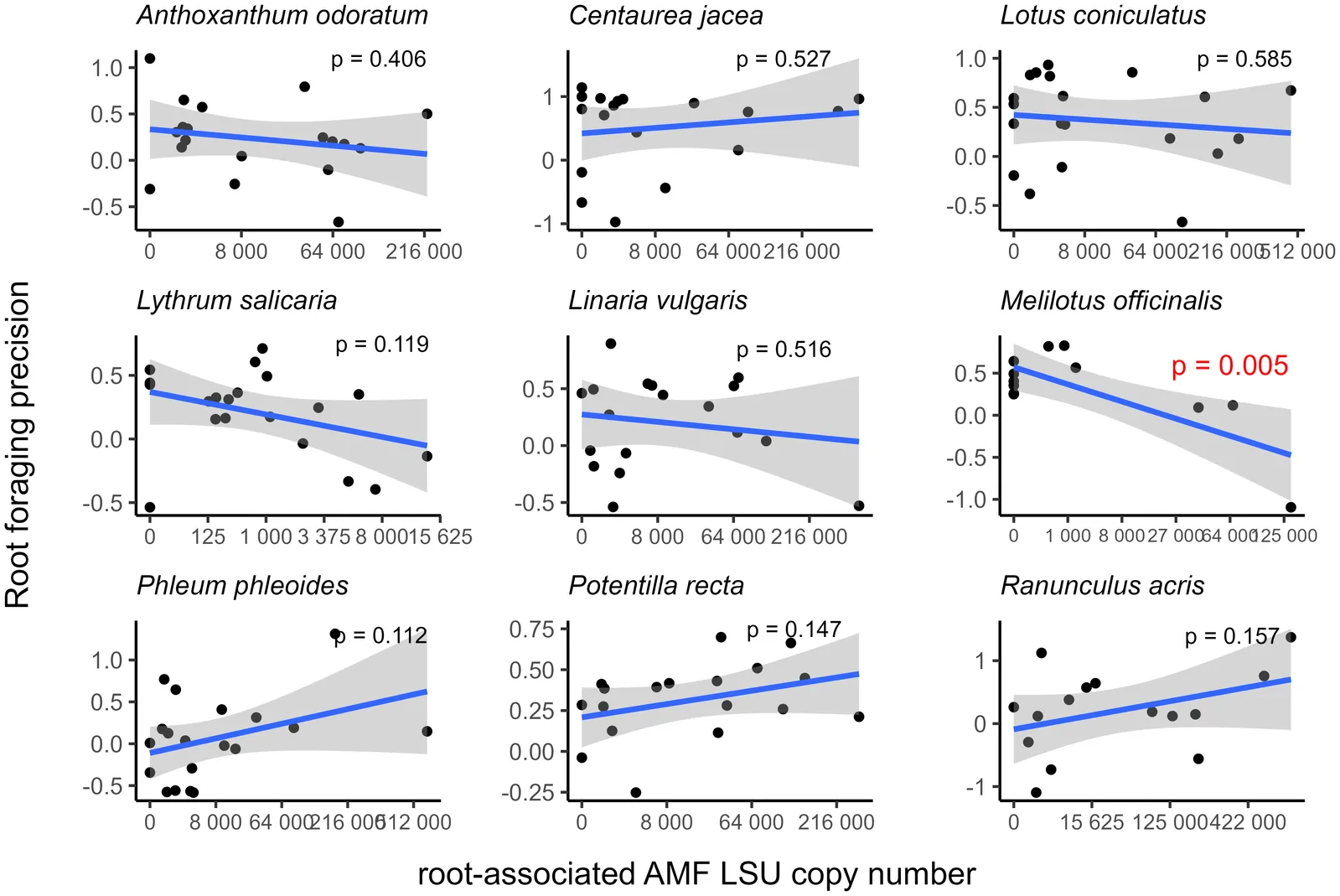
The effects of root-associated AMF LSU copy number on the root foraging precision of nine plant species in a heterogeneous environment. We analysed only data from pots with heterogeneous nutrient treatment. Root foraging precision values above zero indicate that plants allocated more roots to the nutrient-rich half of the pot and values below zero indicate that plants allocated more roots to the nutrient-poor half of the pot. Each point represents data from one pot, and the blue line is the regression line with confidence intervals. We found a significant decline in root foraging precision with increasing root-associated AMF LSU copy number only in one species, *Melilotus officinalis*.

Root foraging was affected by the mycorrhiza × species identity interaction when the root-associated AMF LSU copy number was used in the analysis of mycorrhizal treatment (**Table 3**, **Figure 4**). This effect was only marginally significant when we used only mycorrhizal inoculated pots for the analyses (**Supplementary Table 5** in the **Supporting Information**) The root foraging precision of *M. officinalis* was significantly lower in pots with a higher root-associated AMF LSU copy number (F_1_,_10_ = 12.44, p < 0.01, *R²* = 0.554). Other species showed no significant effect.

## 4 Discussion

Most vascular plants form some type of mycorrhizal symbiosis, the majority of the cases being the arbuscular type of mycorrhiza (Brundrett and Tedersoo, 2018). By exchanging nutrients and water for carbon products of photosynthesis, the symbiotic fungi affect the growth and nutrient acquisition strategies of the plant. One such strategy is plant roots’ precise foraging for heterogeneously distributed nutrients (Campbell et al., 1991; Kembel and Cahill, 2005). However, previous experimental evidence of mycorrhizal fungi affecting a plant’s ability to forage precisely for nutrients is contradictory (Cui and Caldwell, 1996a; Farley and Fitter, 1999b; Hodge, 2001; Wijesinghe et al., 2001; Felderer et al., 2013). In this study, we addressed the question of whether presence of mycorrhizal inoculum and root-associated AMF LSU copy number affected plant foraging precision by testing nine plant species in heterogeneous soil with or without mycorrhizal fungi.

### 4.1 Root foraging precision

In this study, plant species foraged for soil nutrients with varying degrees of precision as was also shown in previous studies (Campbell et al., 1991; Kembel and Cahill, 2005; Weiser et al., 2016). However, mycorrhizal fungi did not generally affect the root foraging precision of our species selection. The only species affected by mycorrhizal fungi was *M. officinalis*; it showed decreased root foraging precision with higher root-associated AMF LSU copy number in the central part of the root system. In previous studies, the effect of mycorrhizal fungi on root foraging precision was variable: there was either no effect of fungi on root foraging (Farley and Fitter, 1999b; Hodge, 2001; Wijesinghe et al., 2001) or plants showed reduced root foraging precision in association with mycorrhizal fungi (Cui and Caldwell, 1996a; Felderer et al., 2013). The observed reduction in root foraging precision of *M. officinalis* with higher root-associated AMF LSU copy number in the central part of the root system corresponds to the findings of studies on *Lotus japonicus* (Felderer et al., 2013) and *Agropyron desertorum* (Cui and Caldwell, 1996a). These studies showed a decrease in root foraging only in phosphorus (P) patches, whilst we investigated foraging for a mixture of nutrients (nitrogen [N], P, potassium [K] + micronutrients). As a leguminous species, *M. officinalis* is limited mainly by P availability in the soil, since it obtains most of its N through its association with symbiotic N_2_-fixing bacteria. Therefore, even though we tested foraging responses to all three major nutrients (N, P, and K), *M. officinalis* could have been limited only (or mainly) by the P. Consequently, if mycorrhizal fungi contributed to P acquisition of *M. officinalis*, the plant may have had sufficient amounts of all essential nutrients and could reduce its root foraging activity. In contrast, other species in the study might have remained limited by N and thus continued to forage even when associated with mycorrhizal fungi. However, we did not directly measure nodulation by nitrogen-fixing bacteria, nitrogen fixation activity, plant tissue N:P ratios, or responses to isolated N, P, or K patches here. Moreover, the second leguminous species in the study, *L. corniculatus*, did not exhibit the same trend as *M. officinalis*; it did not show a change in root foraging precision after mycorrhizal inoculation. Thus, it remains unclear whether the pattern observed in *M. officinalis* reflected P limitation resulting from the association with nitrogen-fixing bacteria, and this hypothesis requires further investigation.

These contrasting results raise a question that has not yet been fully answered: Which nutrients are plant species actually foraging for? Previous studies have shown that some species forage with varying degrees of precision for major nutrients, particularly N, P and K (Drew, 1975; Campbell et al., 1991), as well as specifically for ammonium (Drew, 1975), calcium and magnesium (Skálová et al., 2025). The use of a nutrient mixture in our experiment helped to overcome this uncertainty; however, it came at the cost of a more complex interpretation of the results, as it did not allow us to disentangle foraging responses to individual nutrients.

In soil, N is usually more abundant and mobile than P (Farley and Fitter, 1999a). Therefore, plants are often able to acquire N effectively on their own, without the assistance of AMF, which they rely on primarily for P uptake (Smith et al., 2011). One possible explanation for our results is that, in nutrient patches containing a mixture of nutrients, plants may have responded primarily to N availability, whilst AMF may have been more strongly associated with P acquisition. This might explain why we did not observe a reduction in root foraging precisions, except in *M. officinalis*, despite the observed values of root-associated AMF LSU copy number. However, this remains unclear, as it is not supported by direct measurements of extraradical hyphal development, fungal nutrient uptake, or nutrient transfer to plants. Another possible explanation for the absence of an effect of mycorrhizal inoculation on root foraging precision is the generally lower level of root-associated AMF LSU copy number, similarly to Farley and Fitter (1999b).

This study partially supports previous research (Stiblíková et al., 2023) that linked root-foraging precision to the root economic spectrum but did not find a relationship between foraging precision and mycorrhizal association or other root traits. Under optimal experimental conditions, root-foraging precision appears to be a species-specific trait that could theoretically be positioned along the collaboration gradient of the root economic spectrum (Bergmann et al., 2020). However, a theoretical study involving 123 species (Stiblíková et al., 2023) suggested that root-foraging precision constitutes an independent axis within this spectrum; our results may thus support this conclusion. In our study, when the fungal partner was removed from reportedly mycorrhizal plant species (Guerrero-Ramirez et al., 2021), root-foraging precision generally remained unaffected; only plant biomass was altered. Clearly, additional factors, such as nutrient type or form, could play a role in the allocation between root and fungi.

The balance between allocation of resources (primarily the carbon) to roots or fungal partner may play a role in the natural distribution of plant species over the course of succession as it depends on nutrient availability. Plants are more likely to rely on mycorrhizal hyphae to gain nutrients in nutrient-poor environments, whilst they tend to use roots in nutrient-rich sites (Hoepfner et al., 2015a). Indeed, it is more advantageous for plants to acquire nutrients using mycorrhizal fungi than their own fine roots in sand ecosystems (Hoepfner et al., 2015b). In our study, the nutrient supply was constant and high, which could have led to plants not requiring mycorrhizal symbionts for sufficient nutrient uptake; thus, different results may be obtained under nutrient deficiency.

### 4.2 Plant biomass allocation

Although our main focus was on the effects of mycorrhizal inoculation on root foraging precision, we also analysed plant biomass allocation to better understand plant responses associated with AMF. Biomass declined or did not change as root-associated AMF LSU copy number increased. The effect was similar for both aboveground and belowground biomass. Mycorrhizal symbiosis should not be regarded unequivocally as a mutualistic relationship. Plants’ growth responses may range from positive to neutral or negative, thus the relationship is a continuum from parasitism to mutualism (Johnson et al., 1997). Whether mycorrhizal fungi help plants or parasitise them also depends on the environment and local adaptation (Klironomos, 2003), as well as nutrient and water availability (Mason et al., 2000; Lekberg and Koide, 2008; Frew, 2023), both of which was quite high in our experiment. At least one fungal species, *Rhizophagus irregularis*, has a more mutualistic effect on plant P acquisition under drought conditions (Püschel et al., 2021). We used this species in our study, but it was grown under sufficient moisture.

Interestingly, plant investment in roots compare to shoots in our study did not change under the mycorrhizal or nutrient treatment. The root-associated AMF LSU copy number affected the R:S ratio only in interaction with nutrient treatments; plus, this effect was not apparent in the analyses only with pots to which mycorrhizal inoculum was added – thus without zeroes (corresponding to plants without detectable root-associated AMF LSU copy number) in non-mycorrhizal inoculum. The expected relationship with mutualistic fungi, i.e. higher biomass investment to shoots when the plant has a mycorrhizal partner that helps it to acquire nutrients, occurred in the homogeneous treatment. The effect in the heterogeneous treatment was the opposite, i.e. plants invested more biomass in roots when the root-associated AMF LSU copy number per unit weight of roots was higher, but this effect was rather weak and possibly spurious. We assessed mycorrhizal inoculation using the root-associated AMF LSU copy number per unit root mass in the central part of the root system as a proxy for the presence and abundance of AMF DNA. It does not necessarily indicate mycorrhizal activity outside of this zone and mycorrhiza-mediated nutrient transfer to the host plant. We did not measure arbuscule abundance or extraradical hyphal density; thus, we cannot determine whether differences in root-associated AMF LSU copy number reflected differences in the functional activity of the mycorrhizal fungi.

The reduction in plant biomass in the presence of mycorrhizal fungi in our foraging experiment was also observed by Wijesinghe et al. (2001). In their experiment, the mycorrhizal abundance was fairly low; thus, the effects of fungi would have hardly led to any prominent benefits. They observed higher biomass investment in shoots in the presence of mycorrhiza, but only in species with the highest level of mycorrhizal colonisation. Unlike our study, Wijesinghe et al. (2001) also measured biomass investment in flowers and flowering shoots compared to that in roots. A previous foraging study using the same fungal species as ours (Felderer et al., 2013) observed an increase in plant biomass in the mycorrhizal treatment, but the level of root-associated AMF LSU copy number was much higher compared to that in our experiment. Other previous studies of root foraging under mycorrhizal and non-mycorrhizal treatments also found no change in the R:S ratio in response to the combined mycorrhizal and nutrient treatment.

### 4.3 Limitations

The extent of mycorrhizal inoculation of plant species differed in our study despite giving plant roots and fungal hyphae sufficient time to establish mycorrhizal symbiosis. The number of pots with detectable root-associated AMF LSU copy number ranged from one to all inoculated pots (max. 19, **Supplementary Tables 3** and **7** in the **Supporting Information**) for both plant and fungal species. The root-associated AMF LSU copy number in pots within the same treatment also differed widely. All plant species used in the study are naturally mycorrhizal but their mycorrhizal responsiveness differs (Soudzilovskaia et al., 2020) and, consequently, so may their belowground carbon allocation trade-off, i.e. the allocation to either roots or fungal hyphae, which depends on it (Unger et al., 2016). Moreover, the fungal symbionts we provided might not have suited all plant species, although we used a mixture of three fungal species which imitates natural conditions better than the use of a single fungal species. Usually, most AMF can enter symbiosis with most host species, although specific plant–fungus combinations are probably more profitable than others and affect the growth of plants and fungi differently (Bever et al., 1996; Klironomos, 2003).

Beyond natural factors, the experimental water and temperature regime might have contributed to the relatively low values of detected root-associated AMF LSU copy number, although its influence was not measured and tested. Frequent irrigation (a condition necessary for stable nutrient patch formation) and occasionally high temperatures might have contributed to reduced AMF establishment (Mason et al., 2000; Lekberg and Koide, 2008; Püschel et al., 2021; Frew, 2023; but see Stock et al., 2021). In addition to the suboptimal conditions for the development of mycorrhizal symbiosis, another reason for rather low values of root-associated AMF LSU copy number could be the overall favourable conditions for plants even without mycorrhizal support (enough water and soluble nutrients); thus, the benefits of the symbiosis may have been limited. Absence of strong N and/or P limitation in the substrate may thus have led to decreased allocation by plants to AMF (Johnson et al., 2003), although it should be noted that we did not directly measure nutrient concentrations in either the substrate or the plants. The soil N:P ratio may affect plant investment in AMF, although this may be species-specific depending on the plants’ mycorrhizal responsiveness (Unger et al., 2016).

### 4.4 Conclusion

Our results suggest that root-associated AMF LSU copy number did not consistently affect the root foraging precision of the nine herbaceous plant species we investigated. Although one species (*M. officinalis*) showed reduced root foraging precision with increasing root-associated AMF LSU copy number in the central part of the root system, the remaining species were unaffected, suggesting that the role of mycorrhizal inoculation in root nutrient foraging is neither universal nor particularly common. These results support the view that root foraging precision is a species-specific trait that is not necessarily aligned with the collaboration gradient of the root economic spectrum but instead may represent an independent axis within it (Stiblíková et al., 2023). The removal of the fungal partner did not consistently and strongly affect root foraging precision, indicating that factors such as nutrient type rather than the position of the plant species in the collaboration gradient may influence the observed relationship between root foraging precision and root-associated AMF LSU copy number.

However, our results might also have been affected by variability in root-associated AMF LSU copy number. The growing conditions might have shifted the mycorrhizal symbiosis from mutualistic towards a more parasitic interaction, as indicated by reduced plant biomass at higher levels of root-associated AMF LSU copy number. The relationship between root foraging precision and root-associated AMF LSU copy number likely depends on several factors, including the amount, distribution, and type of nutrients (Hoepfner et al., 2015a; Wang et al., 2016). Future research should investigate how root foraging responses are associated with extraradical hyphal development and nutrient transfer under varying degrees of nutrient limitation, with a focus on the spatial separation of individual nutrients (especially the N and P). A better grasp of these dynamics is essential for predicting plant nutrient acquisition strategies across diverse environmental contexts which helps to understand the root economic spectrum of plants.

## Data Availability

The raw data supporting the conclusions of this article will be made available by the authors, without undue reservation.
