# Supplementary material for: Root-fungal interactions upon resource foraging in heterogeneous soils
Source: Front Plant Sci. 2026 Aug 31;17:1899078. doi: 10.3389/fpls.2026.1899078 (PMC13572630; doi:10.3389/fpls.2026.1899078)
Supplement: Supplementary file 1 [file Table1.docx]

Supplementary Material

# 1 Supplementary Tables and Figures

## 1.1 Supplementary Tables

**Supplementary Table 1.** We tested 24 herbaceous species for germination and mycorrhizal association with our inoculum. Their root foraging precision (**RFP**) was calculated as the decadic logarithm of N/W, where N was the biomass of dried roots from the nutrient-rich half of a given pot and W was the biomass of dried roots from the nutrient-poor half of the same pot (data from our previous experiments, for methodology see Weiser et al. [2016]). The type of mycorrhizal association of the plant species was obtained from the FungalRoot database (Soudzilovskaia et al. [2020]; **mycorrhiza**: AM = arbuscular mycorrhiza). The extent of mycorrhizal colonisation was determined using trypan blue staining (**TBS**; from low = 1 to high = 5, 0 = no colonisation). In the case of germination rate and seedling growth (**germination and growth**), ‘good’ indicated ≥20% of the seeds germinating or ≥60% of seedlings surviving throughout the experiment and ‘bad’ indicated <20% of the seeds germinating or <60% of the seedlings surviving. Mycorrhizal colonisation in the experiment was determined using qPCR (the gene copy numbers of large ribosomal subunit of individual arbuscular mycorrhizal fungal taxa, *Funneliformis mosseae*, *Entrophospora claroidea*, and *Rhizophagus irregularis,* per unit weight of roots summed together (**AMF**); the percentage of ***F. mosseae***, ***R. irregularis***, and ***E. claroidea*** in **AMF**). The species selected for the main experiment are marked in green. Seeds of the 24 tested species (obtained from commercial source Planta Naturalis) were sterilised with a solution of sodium hypochlorite (5% w/w) and water at a ratio of 1:10 for 10 minutes and then washed in demineralised water. We sowed the seeds in Petri dishes containing sterilised sand (autoclaved at 100–120 °C/6 h) and placed the dishes inside a growth chamber (16:8 h day/night cycle with 20/15 °C temperature, respectively) at the end of February 2019. Two weeks later, we transplanted seedlings into 200 mL pots which contained mycorrhizal or non-mycorrhizal inoculum (the same as in the main experiment, see Methods). There were five replicates per each treatment and species combination, i.e. a total of 240 pots. The substrate was a mixture of the inoculum (5%), gamma (25 kGy)-sterilised soil (9%), and autoclaved (at 100–120 °C/6 h) sand (43%) and zeolite (43%). All equipment, including pots, was sterilised with a solution of sodium hypochlorite (5% w/w) and water at a 1:10 ratio. The plants were kept in the greenhouse and watered regularly. We harvested them after five weeks. The roots and shoots were separated, washed, dried (65 °C, 48 h) and weighed. We milled dried roots from four pots from the mycorrhizal treatment and one pot from the non-mycorrhizal treatment for qPCR analyses. The methodologies for sample preparation for qPCR and the analyses are described in the Methods section. The number of pots used for qPCR analyses is given in the last column of this table. We used one pot per treatment for TBS and microscopic determination of mycorrhizal colonisation. If there were not enough pots, root staining was omitted. TBS was performed according to Koske and Gemma (1989), and the percent colonisation of AMF root structures was scored using the gridline intersect approach (McGonigle et al. 1990) at the following scale: **1** = up to 20% colonisation of the root length; **2** = 21%–40% colonisation; **3** = 41%–60% colonisation; **4** = 61%–80% colonisation; **5** = 81%–100% colonisation.

| Species | Family | RFP | Mycorrhiza | TBS | Germination and growth | AMF  (millions of gene copies) | *F. mosseae*  (%) | *R. irregularis*  (%) | *E. claroidea*  (%) | Number of pots for qPCR |
| --- | --- | --- | --- | --- | --- | --- | --- | --- | --- | --- |
| ***Anthoxanthum odoratum*** | ***Poaceae*** | **0.215** | **AM** | **1** | **Good** | **5.51** | **83.5** | **11.5** | **5.0** | **4** |
| ***Centaurea jacea*** | ***Asteraceae*** | **0.416** | **AM** | **1** | **Good** | **16.70** | **91.8** | **6.1** | **2.1** | **3** |
| *Cirsium vulgare* | *Asteraceae* | 0.498 | AM | 1.5 | Good | 15.24 | 56.6 | 41.8 | 1.6 | 4 |
| *Dianthus carthusianorum* | *Caryophyllaceae* | 0.033 | AM | 1 | Good | 7.96 | 66.4 | 22.0 | 11.6 | 4 |
| *Euphorbia esula* | *Euphorbiaceae* | 0.237 | AM | 1 | Bad | NA | NA | NA | NA | NA |
| *Filipendula vulgaris* | *Rosaceae* | 0.179 | AM | 1 | Bad | 6.91 | 37.4 | 52.2 | 10.4 | 4 |
| *Galium verum* | *Rubiaceae* | 0.305 | AM | 1 | Bad | 13.45 | 9.7 | 88.2 | 2.1 | 3 |
| *Geum urbanum* | *Rosaceae* | 0.164 | AM | 5 | Good | 18.97 | 93.2 | 4.4 | 2.4 | 3 |
| *Holcus lanatus* | *Poaceae* | 0.297 | AM | 5 | Good | 0.71 | 60.8 | 37.9 | 1.3 | 4 |
| *Hypochaeris radicata* | *Asteraceae* | 0.475 | AM | 1 | Good | 10.21 | 93.8 | 4.6 | 1.6 | 4 |
| *Koeleria pyramidata* | *Poaceae* | 0.062 | AM | 0 | Good | 39.64 | 95.5 | 0 | 4.5 | 4 |
| ***Linaria vulgaris*** | ***Plantaginaceae*** | **0.203** | **AM** | **0** | **Good** | **1.23** | **78.0** | **16.9** | **5.1** | **4** |
| ***Lotus corniculatus*** | ***Fabaceae*** | **0.361** | **AM** | **5** | **Good** | **12.58** | **92.9** | **5.6** | **1.5** | **4** |
| ***Lythrum salicaria*** | ***Lythraceae*** | **0.568** | **AM** | **0** | **Good** | **7.12** | **85.8** | **12.6** | **1.6** | **4** |
| ***Melilotus officinalis*** | ***Fabaceae*** | **0.596** | **AM** | **1** | **Good** | **8.43** | **96.5** | **2.7** | **0.8** | **3** |
| ***Phleum phleoides*** | ***Poaceae*** | **0.179** | **AM** | **NA** | **Good** | **2.15** | **64.4** | **28.3** | **7.3** | **4** |
| *Plantago lanceolata* | *Plantaginaceae* | 0.237 | AM | NA | Good | 12.58 | 89.5 | 9.3 | 1.2 | 4 |
| ***Potentilla recta*** | ***Rosaceae*** | **0.299** | **AM** | **2** | **Good** | **13.53** | **94.8** | **2.2** | **3.0** | **4** |
| ***Ranunculus acris*** | ***Ranunculaceae*** | **0.182** | **AM** | **5** | **Bad** | **22.01** | **98.0** | **1.2** | **0.8** | **2** |
| *Ranunculus bulbosus* | *Ranunculaceae* | 0.083 | AM | 1.5 | Bad | NA | NA | NA | NA | NA |
| *Tanacetum vulgare* | *Asteraceae* | 0.515 | AM | NA | Good | 8.13 | 98.0 | 1.3 | 0.7 | 4 |
| *Thalictrum minus* | *Ranunculaceae* | 0.328 | AM | NA | Bad | 23.51 | 92.5 | 7.4 | 0.1 | 1 |
| *Trifolium pratense* | *Fabaceae* | 0.203 | AM | NA | Good | 30.72 | 92.9 | 6.2 | 0.9 | 4 |
| *Verbena officinalis* | *Verbenaceae* | 0.352 | AM | 1.5 | Bad | 17.65 | 99.5 | 0.2 | 0.3 | 2 |

**Supplementary Table 2**. The composition of fertiliser (Wuxal Super, Aglukon) used in this study. All nutrients were water soluble and the cationic micronutrients (Fe, Cu, Mn and Zn) were fully chelated with EDTA; pH: 5.5.

| % w/w |  |  | g/l |
| --- | --- | --- | --- |
| 8 | N | Nitrogen | 99.2 |
| 8 | P | Phosphorus | 99.2 |
| 6 | K | Potassium | 74.4 |
| 0.01 | B | Boron | 0.12 |
| 0.004 | Cu | Copper | 0.05 |
| 0.02 | Fe | Iron | 0.25 |
| 0.012 | Mn | Manganese | 0.15 |
| 0.001 | Mo | Molybdenum | 0.012 |
| 0.004 | Zn | Zinc | 0.05 |

**Supplementary Table 3.** This dataset of 323 pots was generated during the experiment. For root biomass, we report only whole root system biomass. The mycorrhizal data, analysed by qPCR using samples from the central part of the roots system (see Methods), are presented in the last three columns, labelled according to the fungal species: *Funneliformis mosseae*, *Rhizophagus irregularis*, and *Entrophospora claroidea*. The qPCR results (Cq values) were converted to root-associated AMF LSU copy number of the individual AMF taxa per unit weight of roots and corrected for extraction efficiency based on the recovery of the internal standard, following Thonar et al. (2012). A value zero in these three columns indicates no detectable root-associated AMF LSU copy number. One sample belonging to the species *L. salicaria* (highlighted in red) was excluded from the data analyses due to very low DNA recovery, most likely resulting from the small amount of available root material, which rendered the corrected qPCR estimate of mycorrhizal abundance unreliable. Three other samples (also highlighted in red) were excluded from the data analyses due to missing values of root-associated AMF LSU copy number.

| Species | Nutrient treatment | Mycorrhizal treatment | Pot number | Whole root biomass | Shoot biomass | Root foraging precision | *Funneliformis mosseae* | *Rhizophagus irregularis* | *Entrophospora claroidea* |
| --- | --- | --- | --- | --- | --- | --- | --- | --- | --- |
| *Anthoxanthum odoratum* | homogeneous | mycorrhizal | 1 | 0.251 | 1.993 | 0.234 | 26248.95 | 0.00 | 0.00 |
| *Anthoxanthum odoratum* | homogeneous | mycorrhizal | 2 | 0.197 | 1.167 | 0.000 | 1726.82 | 0.00 | 0.00 |
| *Anthoxanthum odoratum* | homogeneous | mycorrhizal | 3 | 0.025 | 0.190 | 0.602 | 575.30 | 0.00 | 0.00 |
| *Anthoxanthum odoratum* | homogeneous | mycorrhizal | 4 | 0.457 | 5.194 | -0.003 | 136854.71 | 0.00 | 0.00 |
| *Anthoxanthum odoratum* | homogeneous | mycorrhizal | 5 | 0.829 | 4.645 | 0.180 | 7870.91 | 20864.75 | 0.00 |
| *Anthoxanthum odoratum* | homogeneous | mycorrhizal | 7 | 0.109 | 0.726 | -0.054 | 16470.49 | 0.00 | 0.00 |
| *Anthoxanthum odoratum* | homogeneous | mycorrhizal | 8 | 0.303 | 3.571 | -0.005 | 1858.00 | 0.00 | 12621.58 |
| *Anthoxanthum odoratum* | homogeneous | mycorrhizal | 9 | 0.367 | 2.007 | 0.318 | 27405.59 | 25577.33 | 30114.68 |
| *Anthoxanthum odoratum* | homogeneous | mycorrhizal | 10 | 0.281 | 1.517 | -0.293 | 1390.34 | 0.00 | 0.00 |
| *Anthoxanthum odoratum* | homogeneous | non-mycorrhizal | 1 | 0.940 | 5.492 | -0.062 | 0.00 | 0.00 | 0.00 |
| *Anthoxanthum odoratum* | homogeneous | non-mycorrhizal | 2 | 0.295 | 3.470 | -0.034 | 9612.54 | 0.00 | 0.00 |
| *Anthoxanthum odoratum* | homogeneous | non-mycorrhizal | 3 | 1.091 | 10.309 | 0.076 | 0.00 | 0.00 | 0.00 |
| *Anthoxanthum odoratum* | homogeneous | non-mycorrhizal | 4 | 0.837 | 6.281 | 0.269 | 303.25 | 0.00 | 0.00 |
| *Anthoxanthum odoratum* | homogeneous | non-mycorrhizal | 6 | 0.425 | 4.615 | 0.307 | 1476.44 | 0.00 | 4872.69 |
| *Anthoxanthum odoratum* | homogeneous | non-mycorrhizal | 7 | 0.889 | 4.183 | 0.052 | 0.00 | 0.00 | 0.00 |
| *Anthoxanthum odoratum* | homogeneous | non-mycorrhizal | 8 | 0.537 | 3.652 | 0.002 | 0.00 | 0.00 | 0.00 |
| *Anthoxanthum odoratum* | homogeneous | non-mycorrhizal | 9 | 0.796 | 5.139 | -0.066 | 0.00 | 0.00 | 0.00 |
| *Anthoxanthum odoratum* | homogeneous | non-mycorrhizal | 10 | 0.185 | 0.822 | -0.341 | 97.44 | 0.00 | 0.00 |
| *Anthoxanthum odoratum* | heterogeneous | mycorrhizal | 1 | 0.692 | 7.953 | 0.793 | 17489.67 | 21218.65 | 0.00 |
| *Anthoxanthum odoratum* | heterogeneous | mycorrhizal | 2 | 0.769 | 2.908 | 0.358 | 372.33 | 0.00 | 0.00 |
| *Anthoxanthum odoratum* | heterogeneous | mycorrhizal | 3 | 0.722 | 4.157 | 0.174 | 66936.13 | 9713.00 | 0.00 |
| *Anthoxanthum odoratum* | heterogeneous | mycorrhizal | 4 | 0.471 | 3.847 | 0.574 | 1492.62 | 0.00 | 0.00 |
| *Anthoxanthum odoratum* | heterogeneous | mycorrhizal | 5 | 0.206 | 2.286 | 0.501 | 143178.52 | 79214.04 | 0.00 |
| *Anthoxanthum odoratum* | heterogeneous | mycorrhizal | 6 | 1.835 | 10.431 | 0.203 | 36339.17 | 27119.48 | 0.00 |
| *Anthoxanthum odoratum* | heterogeneous | mycorrhizal | 7 | 0.315 | 2.789 | -0.102 | 23595.40 | 23194.35 | 12353.84 |
| *Anthoxanthum odoratum* | heterogeneous | mycorrhizal | 8 | 0.692 | 4.353 | 0.246 | 54043.60 | 0.00 | 0.00 |
| *Anthoxanthum odoratum* | heterogeneous | mycorrhizal | 9 | 0.452 | 8.231 | 0.130 | 87997.75 | 6969.51 | 2548.20 |
| *Anthoxanthum odoratum* | heterogeneous | mycorrhizal | 10 | 0.727 | 5.002 | -0.255 | 6367.96 | 0.00 | 0.00 |
| *Anthoxanthum odoratum* | heterogeneous | non-mycorrhizal | 1 | 0.468 | 2.733 | 0.304 | 200.40 | 0.00 | 0.00 |
| *Anthoxanthum odoratum* | heterogeneous | non-mycorrhizal | 2 | 0.152 | 1.201 | 1.098 | 0.00 | 0.00 | 0.00 |
| *Anthoxanthum odoratum* | heterogeneous | non-mycorrhizal | 4 | 0.749 | 6.950 | -0.310 | 0.00 | 0.00 | 0.00 |
| *Anthoxanthum odoratum* | heterogeneous | non-mycorrhizal | 5 | 0.734 | 4.695 | 0.341 | 555.72 | 0.00 | 0.00 |
| *Anthoxanthum odoratum* | heterogeneous | non-mycorrhizal | 6 | 0.804 | 7.986 | -0.665 | 27704.71 | 42319.95 | 0.00 |
| *Anthoxanthum odoratum* | heterogeneous | non-mycorrhizal | 7 | 0.237 | 2.360 | 0.651 | 407.59 | 0.00 | 0.00 |
| *Anthoxanthum odoratum* | heterogeneous | non-mycorrhizal | 8 | 0.324 | 4.305 | 0.140 | 333.47 | 0.00 | 0.00 |
| *Anthoxanthum odoratum* | heterogeneous | non-mycorrhizal | 9 | 0.223 | 2.358 | 0.045 | 8067.54 | 0.00 | 0.00 |
| *Anthoxanthum odoratum* | heterogeneous | non-mycorrhizal | 10 | 0.194 | 0.867 | 0.216 | 471.14 | 0.00 | 0.00 |
| *Centaurea jacea* | homogeneous | mycorrhizal | 1 | 1.981 | 14.046 | -0.421 | 72658.44 | 108378.77 | 0.00 |
| *Centaurea jacea* | homogeneous | mycorrhizal | 2 | 0.709 | 5.172 | -0.052 | 38850.60 | 836423.91 | 10423.22 |
| *Centaurea jacea* | homogeneous | mycorrhizal | 3 | 0.880 | 5.237 | -0.267 | 40856.82 | 64146.30 | 0.00 |
| *Centaurea jacea* | homogeneous | mycorrhizal | 4 | 0.957 | 8.217 | -0.133 | 105067.96 | 30904.90 | 0.00 |
| *Centaurea jacea* | homogeneous | mycorrhizal | 5 | 2.201 | 17.977 | 0.119 | 114219.99 | 181659.94 | 2158.04 |
| *Centaurea jacea* | homogeneous | mycorrhizal | 6 | 1.548 | 12.471 | 0.091 | 131952.17 | 490661.87 | 213580.08 |
| *Centaurea jacea* | homogeneous | mycorrhizal | 7 | 0.556 | 6.926 | -0.313 | 10407.97 | 19882.21 | 0.00 |
| *Centaurea jacea* | homogeneous | mycorrhizal | 8 | 1.677 | 13.079 | -0.085 | 5086.45 | 66506.16 | 0.00 |
| *Centaurea jacea* | homogeneous | mycorrhizal | 9 | 1.728 | 12.865 | -0.764 | 2974.67 | 0.00 | 0.00 |
| *Centaurea jacea* | homogeneous | mycorrhizal | 10 | 0.984 | 12.482 | 0.202 | 133406.21 | 303487.29 | 9282.89 |
| *Centaurea jacea* | homogeneous | non-mycorrhizal | 1 | 1.258 | 11.650 | 0.234 | 8589.58 | 0.00 | 0.00 |
| *Centaurea jacea* | homogeneous | non-mycorrhizal | 2 | 1.211 | 12.109 | 0.137 | 370.99 | 0.00 | 0.00 |
| *Centaurea jacea* | homogeneous | non-mycorrhizal | 3 | 0.438 | 5.005 | 0.094 | 0.00 | 0.00 | 0.00 |
| *Centaurea jacea* | homogeneous | non-mycorrhizal | 4 | 1.128 | 8.633 | -0.194 | 221.03 | 0.00 | 0.00 |
| *Centaurea jacea* | homogeneous | non-mycorrhizal | 5 | 0.568 | 12.609 | 0.413 | 45013.56 | 68573.48 | 0.00 |
| *Centaurea jacea* | homogeneous | non-mycorrhizal | 6 | 1.674 | 10.909 | 0.321 | 0.00 | 0.00 | 0.00 |
| *Centaurea jacea* | homogeneous | non-mycorrhizal | 7 | 0.640 | 6.364 | 0.222 | 0.00 | 0.00 | 0.00 |
| *Centaurea jacea* | homogeneous | non-mycorrhizal | 8 | 1.000 | 10.597 | 0.290 | 303.61 | 0.00 | 0.00 |
| *Centaurea jacea* | homogeneous | non-mycorrhizal | 9 | 1.351 | 7.152 | -0.396 | 0.00 | 0.00 | 0.00 |
| *Centaurea jacea* | homogeneous | non-mycorrhizal | 10 | 2.171 | 21.603 | 0.137 | 2333.89 | 0.00 | 0.00 |
| *Centaurea jacea* | heterogeneous | mycorrhizal | 1 | 0.847 | 17.169 | 0.960 | 1406.65 | 0.00 | 0.00 |
| *Centaurea jacea* | heterogeneous | mycorrhizal | 2 | 2.530 | 22.478 | 0.758 | 6412.42 | 86474.11 | 0.00 |
| *Centaurea jacea* | heterogeneous | mycorrhizal | 3 | 1.523 | 24.300 | 0.158 | 23333.34 | 54087.46 | 0.00 |
| *Centaurea jacea* | heterogeneous | mycorrhizal | 4 | 2.241 | 27.590 | 0.861 | 654.58 | 0.00 | 0.00 |
| *Centaurea jacea* | heterogeneous | mycorrhizal | 5 | 0.703 | 8.872 | 0.771 | 138108.65 | 202125.75 | 0.00 |
| *Centaurea jacea* | heterogeneous | mycorrhizal | 6 | 2.289 | 10.479 | 0.963 | 58191.39 | 368894.36 | 3559.29 |
| *Centaurea jacea* | heterogeneous | mycorrhizal | 7 | 0.454 | 4.183 | -0.973 | 756.84 | 0.00 | 0.00 |
| *Centaurea jacea* | heterogeneous | mycorrhizal | 8 | 1.726 | 16.502 | 0.897 | 15997.82 | 12579.50 | 0.00 |
| *Centaurea jacea* | heterogeneous | mycorrhizal | 10 | 0.838 | 9.046 | 0.439 | 3315.38 | 0.00 | 0.00 |
| *Centaurea jacea* | heterogeneous | non-mycorrhizal | 1 | 2.134 | 20.833 | 0.708 | 223.92 | 0.00 | 0.00 |
| *Centaurea jacea* | heterogeneous | non-mycorrhizal | 3 | 0.952 | 8.551 | -0.666 | 0.00 | 0.00 | 0.00 |
| *Centaurea jacea* | heterogeneous | non-mycorrhizal | 4 | 0.411 | 3.759 | 0.928 | 935.58 | 0.00 | 0.00 |
| *Centaurea jacea* | heterogeneous | non-mycorrhizal | 5 | 1.286 | 8.766 | 1.000 | 0.00 | 0.00 | 0.00 |
| *Centaurea jacea* | heterogeneous | non-mycorrhizal | 6 | 1.849 | 19.390 | 0.973 | 130.96 | 0.00 | 0.00 |
| *Centaurea jacea* | heterogeneous | non-mycorrhizal | 7 | 2.891 | 39.250 | 1.142 | 0.00 | 0.00 | 0.00 |
| *Centaurea jacea* | heterogeneous | non-mycorrhizal | 8 | 1.759 | 24.766 | -0.438 | 11776.18 | 0.00 | 0.00 |
| *Centaurea jacea* | heterogeneous | non-mycorrhizal | 9 | 3.094 | 14.777 | 0.803 | 0.00 | 0.00 | 0.00 |
| *Centaurea jacea* | heterogeneous | non-mycorrhizal | 10 | 0.170 | 1.691 | -0.191 | 0.00 | 0.00 | 0.00 |
| *Lotus corniculatus* | homogeneous | mycorrhizal | 2 | 0.535 | 9.911 | 0.522 | 5358.99 | 12539.83 | 0.00 |
| *Lotus corniculatus* | homogeneous | mycorrhizal | 3 | 0.402 | 8.418 | -0.064 | 162173.01 | 39201.75 | 0.00 |
| *Lotus corniculatus* | homogeneous | mycorrhizal | 4 | 0.463 | 8.876 | 0.168 | 46563.44 | 35927.43 | 0.00 |
| *Lotus corniculatus* | homogeneous | mycorrhizal | 6 | 1.578 | 23.760 | 0.059 | 20325.54 | 0.00 | 0.00 |
| *Lotus corniculatus* | homogeneous | mycorrhizal | 7 | 0.839 | 12.576 | 0.063 | 16126.54 | 0.00 | 0.00 |
| *Lotus corniculatus* | homogeneous | mycorrhizal | 8 | 0.317 | 9.170 | -0.554 | 3090.99 | 0.00 | 0.00 |
| *Lotus corniculatus* | homogeneous | mycorrhizal | 9 | 0.465 | 29.506 | -0.544 | 689.81 | 0.00 | 0.00 |
| *Lotus corniculatus* | homogeneous | mycorrhizal | 10 | 0.654 | 9.515 | -0.116 | 5485.99 | 48197.82 | 0.00 |
| *Lotus corniculatus* | homogeneous | non-mycorrhizal | 1 | 0.005 | NA | -0.566 | 0.00 | 0.00 | 0.00 |
| *Lotus corniculatus* | homogeneous | non-mycorrhizal | 2 | 0.570 | 8.646 | -0.607 | 0.00 | 0.00 | 0.00 |
| *Lotus corniculatus* | homogeneous | non-mycorrhizal | 3 | 0.042 | 0.845 | -1.240 | 0.00 | 120181.00 | 0.00 |
| *Lotus corniculatus* | homogeneous | non-mycorrhizal | 4 | 0.248 | 6.816 | 0.257 | 0.00 | 0.00 | 0.00 |
| *Lotus corniculatus* | homogeneous | non-mycorrhizal | 5 | 1.058 | 14.000 | 0.122 | 4084.92 | 0.00 | 0.00 |
| *Lotus corniculatus* | homogeneous | non-mycorrhizal | 6 | 0.599 | 10.098 | -0.242 | 0.00 | 0.00 | 0.00 |
| *Lotus corniculatus* | homogeneous | non-mycorrhizal | 7 | 0.507 | 6.059 | 0.383 | 0.00 | 0.00 | 0.00 |
| *Lotus corniculatus* | homogeneous | non-mycorrhizal | 8 | 0.235 | 5.878 | -0.468 | 3666.53 | 0.00 | 0.00 |
| *Lotus corniculatus* | homogeneous | non-mycorrhizal | 9 | 0.732 | 13.642 | -0.477 | 0.00 | 0.00 | 0.00 |
| *Lotus corniculatus* | homogeneous | non-mycorrhizal | 10 | 0.907 | 16.184 | -0.182 | 168.21 | 0.00 | 968.80 |
| *Lotus corniculatus* | heterogeneous | mycorrhizal | 1 | 0.695 | 8.843 | 0.932 | 914.19 | 0.00 | 0.00 |
| *Lotus corniculatus* | heterogeneous | mycorrhizal | 2 | 0.440 | 10.313 | 0.184 | 10297.73 | 74950.75 | 0.00 |
| *Lotus corniculatus* | heterogeneous | mycorrhizal | 3 | 0.185 | 3.020 | -0.109 | 2494.09 | 0.00 | 0.00 |
| *Lotus corniculatus* | heterogeneous | mycorrhizal | 4 | 0.125 | 1.977 | 0.336 | 2374.99 | 0.00 | 0.00 |
| *Lotus corniculatus* | heterogeneous | mycorrhizal | 5 | 0.667 | 9.241 | 0.606 | 112758.74 | 43334.71 | 0.00 |
| *Lotus corniculatus* | heterogeneous | mycorrhizal | 6 | 0.383 | 6.737 | -0.668 | 32696.18 | 74100.09 | 0.00 |
| *Lotus corniculatus* | heterogeneous | mycorrhizal | 7 | 1.362 | 16.740 | 0.856 | 10793.03 | 26428.78 | 0.00 |
| *Lotus corniculatus* | heterogeneous | mycorrhizal | 8 | 1.446 | 20.726 | 0.673 | 183404.99 | 293030.73 | 0.00 |
| *Lotus corniculatus* | heterogeneous | mycorrhizal | 9 | 0.510 | 2.591 | 0.180 | 80242.43 | 167308.67 | 6705.80 |
| *Lotus corniculatus* | heterogeneous | mycorrhizal | 10 | 1.491 | 18.242 | 0.029 | 65089.35 | 124672.57 | 0.00 |
| *Lotus corniculatus* | heterogeneous | non-mycorrhizal | 1 | 0.857 | 14.095 | 0.830 | 97.96 | 0.00 | 0.00 |
| *Lotus corniculatus* | heterogeneous | non-mycorrhizal | 2 | 0.086 | 2.793 | -0.194 | 0.00 | 0.00 | 0.00 |
| *Lotus corniculatus* | heterogeneous | non-mycorrhizal | 3 | 0.819 | 12.797 | 0.818 | 1051.57 | 0.00 | 0.00 |
| *Lotus corniculatus* | heterogeneous | non-mycorrhizal | 4 | 0.812 | 15.218 | 0.534 | 0.00 | 0.00 | 0.00 |
| *Lotus corniculatus* | heterogeneous | non-mycorrhizal | 5 | 0.232 | 5.169 | -0.381 | 92.74 | 0.00 | 0.00 |
| *Lotus corniculatus* | heterogeneous | non-mycorrhizal | 6 | 0.707 | 13.054 | 0.615 | 2670.15 | 0.00 | 0.00 |
| *Lotus corniculatus* | heterogeneous | non-mycorrhizal | 7 | 0.271 | 4.496 | 0.593 | 0.00 | 0.00 | 0.00 |
| *Lotus corniculatus* | heterogeneous | non-mycorrhizal | 8 | 0.287 | 2.256 | 0.326 | 499.90 | 0.00 | 2509.98 |
| *Lotus corniculatus* | heterogeneous | non-mycorrhizal | 9 | 0.467 | 13.983 | 0.334 | 0.00 | 0.00 | 0.00 |
| *Lotus corniculatus* | heterogeneous | non-mycorrhizal | 10 | 0.696 | 10.952 | 0.855 | 248.87 | 0.00 | 0.00 |
| *Lythrum salicaria* | homogeneous | mycorrhizal | 1 | 1.736 | 18.658 | 0.193 | 332.37 | 0.00 | 0.00 |
| *Lythrum salicaria* | homogeneous | mycorrhizal | 2 | 0.842 | 6.567 | 0.061 | 68.66 | 0.00 | 0.00 |
| *Lythrum salicaria* | homogeneous | mycorrhizal | 3 | 2.617 | 22.539 | 0.099 | 82.06 | 0.00 | 0.00 |
| *Lythrum salicaria* | homogeneous | mycorrhizal | 4 | 2.280 | 17.283 | 0.209 | 1542.17 | 0.00 | 0.00 |
| *Lythrum salicaria* | homogeneous | mycorrhizal | 5 | 1.869 | 16.421 | 0.150 | 20088.21 | 8716.70 | 0.00 |
| *Lythrum salicaria* | homogeneous | mycorrhizal | 6 | 5.811 | 36.666 | -0.045 | 1596.52 | 0.00 | 0.00 |
| *Lythrum salicaria* | homogeneous | mycorrhizal | 7 | 2.083 | 19.053 | -0.099 | 24780.13 | 0.00 | 0.00 |
| *Lythrum salicaria* | homogeneous | mycorrhizal | 8 | 0.052 | 0.177 | -0.432 | 4824257.69 | 2757056.71 | 144769.44 |
| *Lythrum salicaria* | homogeneous | mycorrhizal | 9 | 4.099 | 31.947 | 0.021 | 6771.72 | 0.00 | 0.00 |
| *Lythrum salicaria* | homogeneous | mycorrhizal | 10 | 1.094 | 16.210 | 0.151 | 1680.87 | 0.00 | 299.67 |
| *Lythrum salicaria* | homogeneous | non-mycorrhizal | 1 | 3.588 | 18.891 | -0.182 | 2302.78 | 0.00 | 0.00 |
| *Lythrum salicaria* | homogeneous | non-mycorrhizal | 2 | 1.006 | 20.457 | 0.037 | 11120.59 | 10998.63 | 0.00 |
| *Lythrum salicaria* | homogeneous | non-mycorrhizal | 3 | 4.450 | 28.563 | -0.564 | 1259.12 | 0.00 | 0.00 |
| *Lythrum salicaria* | homogeneous | non-mycorrhizal | 4 | 1.715 | 4.449 | -0.113 | 355.35 | 0.00 | 0.00 |
| *Lythrum salicaria* | homogeneous | non-mycorrhizal | 5 | 6.297 | 37.699 | 0.106 | 194.28 | 0.00 | 0.00 |
| *Lythrum salicaria* | homogeneous | non-mycorrhizal | 6 | 3.802 | 20.456 | 0.036 | 39.26 | 0.00 | 0.00 |
| *Lythrum salicaria* | homogeneous | non-mycorrhizal | 7 | 3.638 | 28.563 | -0.087 | 513.35 | 0.00 | 0.00 |
| *Lythrum salicaria* | homogeneous | non-mycorrhizal | 8 | 0.534 | 4.449 | 0.082 | 70.08 | 0.00 | 0.00 |
| *Lythrum salicaria* | homogeneous | non-mycorrhizal | 9 | 4.577 | 27.671 | 0.010 | 2172.98 | 0.00 | 0.00 |
| *Lythrum salicaria* | homogeneous | non-mycorrhizal | 10 | 2.871 | 40.831 | -0.107 | 5016.16 | 0.00 | 0.00 |
| *Lythrum salicaria* | heterogeneous | mycorrhizal | 1 | 4.570 | 40.386 | -0.333 | 4994.75 | 0.00 | 0.00 |
| *Lythrum salicaria* | heterogeneous | mycorrhizal | 2 | 3.073 | 22.657 | -0.136 | 13600.16 | 0.00 | 0.00 |
| *Lythrum salicaria* | heterogeneous | mycorrhizal | 3 | 2.926 | 21.592 | 0.605 | 743.85 | 0.00 | 0.00 |
| *Lythrum salicaria* | heterogeneous | mycorrhizal | 4 | 2.643 | 24.303 | 0.311 | 310.73 | 0.00 | 0.00 |
| *Lythrum salicaria* | heterogeneous | mycorrhizal | 5 | 4.325 | 29.841 | 0.351 | 5823.21 | 0.00 | 0.00 |
| *Lythrum salicaria* | heterogeneous | mycorrhizal | 6 | 2.453 | 16.745 | 0.164 | 275.24 | 0.00 | 0.00 |
| *Lythrum salicaria* | heterogeneous | mycorrhizal | 7 | 2.932 | 24.160 | -0.396 | 7320.69 | 0.00 | 0.00 |
| *Lythrum salicaria* | heterogeneous | mycorrhizal | 8 | 2.147 | 17.304 | 0.493 | 1023.97 | 0.00 | 0.00 |
| *Lythrum salicaria* | heterogeneous | mycorrhizal | 9 | 2.836 | 27.830 | 0.246 | 3058.51 | 0.00 | 0.00 |
| *Lythrum salicaria* | heterogeneous | mycorrhizal | 10 | 2.138 | 23.992 | 0.155 | 180.27 | 0.00 | 0.00 |
| *Lythrum salicaria* | heterogeneous | non-mycorrhizal | 1 | 0.260 | 1.941 | 0.324 | 184.63 | 0.00 | 0.00 |
| *Lythrum salicaria* | heterogeneous | non-mycorrhizal | 2 | 1.702 | 16.060 | -0.537 | 0.00 | 0.00 | 0.00 |
| *Lythrum salicaria* | heterogeneous | non-mycorrhizal | 3 | 1.706 | 12.826 | -0.035 | 2287.07 | 0.00 | 0.00 |
| *Lythrum salicaria* | heterogeneous | non-mycorrhizal | 4 | 4.033 | 19.118 | 0.174 | 1108.80 | 0.00 | 0.00 |
| *Lythrum salicaria* | heterogeneous | non-mycorrhizal | 5 | 3.865 | 32.267 | 0.363 | 430.64 | 0.00 | 0.00 |
| *Lythrum salicaria* | heterogeneous | non-mycorrhizal | 6 | 2.630 | 22.337 | 0.544 | 0.00 | 0.00 | 0.00 |
| *Lythrum salicaria* | heterogeneous | non-mycorrhizal | 7 | 5.255 | 31.755 | 0.428 | 0.00 | 0.00 | 0.00 |
| *Lythrum salicaria* | heterogeneous | non-mycorrhizal | 8 | 1.436 | 11.911 | 0.712 | 911.25 | 0.00 | 0.00 |
| *Lythrum salicaria* | heterogeneous | non-mycorrhizal | 9 | 3.478 | 26.858 | 0.296 | 129.42 | 0.00 | 0.00 |
| *Lythrum salicaria* | heterogeneous | non-mycorrhizal | 10 | 3.840 | 19.486 | 0.441 | 0.00 | 0.00 | 0.00 |
| *Linaria vulgaris* | homogeneous | mycorrhizal | 2 | 0.084 | 1.541 | 0.562 | 350048.59 | 16900.43 | 10363.69 |
| *Linaria vulgaris* | homogeneous | mycorrhizal | 3 | 0.129 | 1.007 | 0.056 | 548510.63 | 101894.36 | 14875.80 |
| *Linaria vulgaris* | homogeneous | mycorrhizal | 4 | 0.531 | 4.977 | -0.288 | 52375.19 | 0.00 | 0.00 |
| *Linaria vulgaris* | homogeneous | mycorrhizal | 5 | 0.145 | 2.967 | 0.516 | 16937.89 | 0.00 | 0.00 |
| *Linaria vulgaris* | homogeneous | mycorrhizal | 6 | 0.229 | 3.256 | -0.063 | 26828.11 | 0.00 | 0.00 |
| *Linaria vulgaris* | homogeneous | mycorrhizal | 7 | 0.107 | 1.068 | 0.188 | 0.00 | 0.00 | 0.00 |
| *Linaria vulgaris* | homogeneous | mycorrhizal | 9 | 0.042 | 0.343 | 1.048 | 337.68 | 0.00 | 0.00 |
| *Linaria vulgaris* | homogeneous | mycorrhizal | 10 | 0.475 | 4.777 | -0.377 | 244057.31 | 18003.84 | 23315.32 |
| *Linaria vulgaris* | homogeneous | non-mycorrhizal | 1 | 0.039 | 0.476 | -0.365 | 0.00 | 0.00 | 0.00 |
| *Linaria vulgaris* | homogeneous | non-mycorrhizal | 2 | 0.319 | 3.521 | 0.280 | 125.62 | 0.00 | 0.00 |
| *Linaria vulgaris* | homogeneous | non-mycorrhizal | 4 | 0.346 | 4.496 | 0.265 | 88.20 | 0.00 | 0.00 |
| *Linaria vulgaris* | homogeneous | non-mycorrhizal | 5 | 0.479 | 3.767 | 0.781 | 214.39 | 0.00 | 0.00 |
| *Linaria vulgaris* | homogeneous | non-mycorrhizal | 6 | 0.415 | 4.444 | -0.547 | 0.00 | 0.00 | 0.00 |
| *Linaria vulgaris* | homogeneous | non-mycorrhizal | 7 | 0.379 | 5.781 | -0.228 | 109.59 | 0.00 | 0.00 |
| *Linaria vulgaris* | homogeneous | non-mycorrhizal | 8 | 0.345 | 3.574 | 0.061 | 269.60 | 0.00 | 0.00 |
| *Linaria vulgaris* | homogeneous | non-mycorrhizal | 9 | 1.132 | 10.062 | -0.063 | 121.89 | 0.00 | 0.00 |
| *Linaria vulgaris* | homogeneous | non-mycorrhizal | 10 | 0.224 | 2.909 | 0.149 | 9435.89 | 13741.12 | 0.00 |
| *Linaria vulgaris* | heterogeneous | mycorrhizal | 1 | 0.550 | 3.564 | 0.115 | 147680.28 | 83168.77 | 2186.62 |
| *Linaria vulgaris* | heterogeneous | mycorrhizal | 2 | 0.094 | 1.399 | -0.539 | 1888.36 | 0.00 | 0.00 |
| *Linaria vulgaris* | heterogeneous | mycorrhizal | 3 | 0.222 | 3.063 | 0.597 | 173654.89 | 63949.06 | 0.00 |
| *Linaria vulgaris* | heterogeneous | mycorrhizal | 4 | 0.282 | 2.471 | 0.524 | 181930.46 | 30948.10 | 4644.35 |
| *Linaria vulgaris* | heterogeneous | mycorrhizal | 5 | 0.736 | 9.391 | 0.528 | 19923.65 | 0.00 | 1411.87 |
| *Linaria vulgaris* | heterogeneous | mycorrhizal | 6 | 0.008 | 0.088 | 0.040 | 65105.36 | 314764.17 | 7807.65 |
| *Linaria vulgaris* | heterogeneous | mycorrhizal | 7 | 0.206 | 35.744 | 0.445 | 29068.98 | 0.00 | 3998.88 |
| *Linaria vulgaris* | heterogeneous | mycorrhizal | 8 | 0.432 | 3.482 | -0.529 | 61660.85 | 1255823.36 | 0.00 |
| *Linaria vulgaris* | heterogeneous | mycorrhizal | 9 | 0.139 | 0.559 | 0.344 | 72869.69 | 53499.52 | 0.00 |
| *Linaria vulgaris* | heterogeneous | mycorrhizal | 10 | 0.189 | 3.368 | 0.544 | 17736.35 | 0.00 | 0.00 |
| *Linaria vulgaris* | heterogeneous | non-mycorrhizal | 1 | 0.202 | 1.408 | -0.066 | 0.00 | 0.00 | 5321.25 |
| *Linaria vulgaris* | heterogeneous | non-mycorrhizal | 2 | 0.703 | 5.346 | 0.271 | 1264.67 | 0.00 | 0.00 |
| *Linaria vulgaris* | heterogeneous | non-mycorrhizal | 3 | 0.180 | 2.048 | 0.896 | 1509.95 | 0.00 | 0.00 |
| *Linaria vulgaris* | heterogeneous | non-mycorrhizal | 4 | 0.583 | 9.335 | -0.182 | 108.16 | 0.00 | 0.00 |
| *Linaria vulgaris* | heterogeneous | non-mycorrhizal | 5 | 0.558 | 2.109 | -0.241 | 3359.99 | 0.00 | 0.00 |
| *Linaria vulgaris* | heterogeneous | non-mycorrhizal | 6 | 0.633 | 6.416 | 0.494 | 96.58 | 0.00 | 0.00 |
| *Linaria vulgaris* | heterogeneous | non-mycorrhizal | 9 | 0.147 | 1.793 | -0.044 | 37.34 | 0.00 | 0.00 |
| *Linaria vulgaris* | heterogeneous | non-mycorrhizal | 10 | 0.294 | 3.074 | 0.460 | 0.00 | 0.00 | 0.00 |
| *Melilotus officinalis* | homogeneous | mycorrhizal | 1 | 1.201 | 9.234 | -0.463 | 285.42 | 0.00 | 0.00 |
| *Melilotus officinalis* | homogeneous | mycorrhizal | 2 | 5.563 | 26.518 | -0.080 | 10834.37 | 0.00 | 0.00 |
| *Melilotus officinalis* | homogeneous | mycorrhizal | 3 | 5.434 | 22.326 | 0.016 | 122.37 | 0.00 | 0.00 |
| *Melilotus officinalis* | homogeneous | mycorrhizal | 4 | 3.279 | 13.624 | -0.184 | 79.17 | 0.00 | 0.00 |
| *Melilotus officinalis* | homogeneous | mycorrhizal | 5 | 0.583 | 3.359 | -0.425 | 10715.16 | 0.00 | 0.00 |
| *Melilotus officinalis* | homogeneous | mycorrhizal | 6 | NA | 2.518 | -0.585 | NA | NA | NA |
| *Melilotus officinalis* | homogeneous | mycorrhizal | 8 | 0.403 | 3.185 | -0.247 | 1864.19 | 15442.13 | 0.00 |
| *Melilotus officinalis* | homogeneous | mycorrhizal | 9 | 4.231 | 19.206 | -0.448 | 6473.12 | 177167.01 | 4436.96 |
| *Melilotus officinalis* | homogeneous | mycorrhizal | 10 | 1.901 | 16.784 | 0.203 | 0.00 | 0.00 | 0.00 |
| *Melilotus officinalis* | homogeneous | non-mycorrhizal | 1 | 4.925 | 21.718 | -0.231 | 0.00 | 0.00 | 0.00 |
| *Melilotus officinalis* | homogeneous | non-mycorrhizal | 3 | 2.815 | 15.137 | 0.141 | 0.00 | 0.00 | 0.00 |
| *Melilotus officinalis* | homogeneous | non-mycorrhizal | 4 | 5.083 | 20.093 | -0.007 | 0.00 | 0.00 | 0.00 |
| *Melilotus officinalis* | homogeneous | non-mycorrhizal | 5 | NA | 28.658 | 0.101 | 0.00 | 0.00 | 0.00 |
| *Melilotus officinalis* | homogeneous | non-mycorrhizal | 6 | 1.414 | 6.352 | 0.049 | 425.67 | 0.00 | 0.00 |
| *Melilotus officinalis* | homogeneous | non-mycorrhizal | 8 | 6.657 | 25.770 | -0.523 | NA | NA | NA |
| *Melilotus officinalis* | homogeneous | non-mycorrhizal | 9 | 2.887 | 16.343 | -0.218 | 0.00 | 0.00 | 0.00 |
| *Melilotus officinalis* | homogeneous | non-mycorrhizal | 10 | 2.470 | 15.221 | -0.062 | 0.00 | 0.00 | 0.00 |
| *Melilotus officinalis* | heterogeneous | mycorrhizal | 2 | 3.592 | 15.215 | 0.819 | 265.29 | 0.00 | 0.00 |
| *Melilotus officinalis* | heterogeneous | mycorrhizal | 4 | 1.674 | 10.329 | 0.117 | 62382.61 | 0.00 | 4190.62 |
| *Melilotus officinalis* | heterogeneous | mycorrhizal | 5 | 0.994 | 7.871 | -1.094 | 15277.32 | 119497.39 | 0.00 |
| *Melilotus officinalis* | heterogeneous | mycorrhizal | 6 | 2.650 | 11.258 | 0.092 | 21735.46 | 18138.95 | 0.00 |
| *Melilotus officinalis* | heterogeneous | mycorrhizal | 7 | 1.977 | 10.656 | 0.566 | 1508.26 | 0.00 | 0.00 |
| *Melilotus officinalis* | heterogeneous | non-mycorrhizal | 1 | 2.756 | 20.392 | 0.493 | 0.00 | 0.00 | 0.00 |
| *Melilotus officinalis* | heterogeneous | non-mycorrhizal | 3 | 4.943 | 20.661 | 0.828 | 815.29 | 0.00 | 0.00 |
| *Melilotus officinalis* | heterogeneous | non-mycorrhizal | 5 | 3.062 | 18.197 | 0.259 | 0.00 | 0.00 | 0.00 |
| *Melilotus officinalis* | heterogeneous | non-mycorrhizal | 6 | 4.581 | 17.739 | 0.251 | 0.00 | 0.00 | 0.00 |
| *Melilotus officinalis* | heterogeneous | non-mycorrhizal | 7 | 4.080 | 15.302 | 0.642 | 0.00 | 0.00 | 0.00 |
| *Melilotus officinalis* | heterogeneous | non-mycorrhizal | 9 | 4.695 | 24.622 | 0.404 | 0.00 | 0.00 | 0.00 |
| *Melilotus officinalis* | heterogeneous | non-mycorrhizal | 10 | 2.384 | 11.155 | 0.354 | 0.00 | 0.00 | 0.00 |
| *Phleum phleoides* | homogeneous | mycorrhizal | 1 | 0.328 | 1.602 | -0.388 | 72498.11 | 25083.34 | 0.00 |
| *Phleum phleoides* | homogeneous | mycorrhizal | 2 | 0.030 | 0.487 | 1.449 | 119117.32 | 84643.93 | 19684.82 |
| *Phleum phleoides* | homogeneous | mycorrhizal | 3 | 0.035 | 0.150 | -0.029 | 1132.19 | 0.00 | 0.00 |
| *Phleum phleoides* | homogeneous | mycorrhizal | 4 | 0.520 | 2.848 | 0.124 | 39359.20 | 7870.76 | 0.00 |
| *Phleum phleoides* | homogeneous | mycorrhizal | 5 | 0.201 | 0.995 | -0.120 | 38403.84 | 12725.31 | 6156.25 |
| *Phleum phleoides* | homogeneous | mycorrhizal | 6 | 0.194 | 0.847 | 0.727 | 0.00 | 0.00 | 0.00 |
| *Phleum phleoides* | homogeneous | mycorrhizal | 7 | 0.224 | 1.162 | -0.093 | 830.59 | 0.00 | 0.00 |
| *Phleum phleoides* | homogeneous | mycorrhizal | 8 | 0.078 | 0.615 | -0.350 | 53933.71 | 17056.39 | 0.00 |
| *Phleum phleoides* | homogeneous | mycorrhizal | 10 | 0.123 | 0.524 | 0.395 | 22009.43 | 0.00 | 813.33 |
| *Phleum phleoides* | homogeneous | non-mycorrhizal | 1 | 0.015 | 0.063 | -0.135 | 147914.34 | 0.00 | 0.00 |
| *Phleum phleoides* | homogeneous | non-mycorrhizal | 2 | 0.172 | 0.728 | 0.376 | 0.00 | 0.00 | 0.00 |
| *Phleum phleoides* | homogeneous | non-mycorrhizal | 3 | 0.119 | 0.507 | 0.003 | 0.00 | 0.00 | 0.00 |
| *Phleum phleoides* | homogeneous | non-mycorrhizal | 4 | 0.101 | 0.687 | 1.173 | 0.00 | 0.00 | 0.00 |
| *Phleum phleoides* | homogeneous | non-mycorrhizal | 6 | 0.051 | 0.056 | 0.327 | 1271.47 | 0.00 | 0.00 |
| *Phleum phleoides* | homogeneous | non-mycorrhizal | 7 | 0.289 | 1.099 | 0.025 | 4466.30 | 0.00 | 0.00 |
| *Phleum phleoides* | homogeneous | non-mycorrhizal | 8 | 0.169 | 0.863 | -0.570 | 699.31 | 0.00 | 0.00 |
| *Phleum phleoides* | homogeneous | non-mycorrhizal | 9 | 0.074 | 0.234 | -0.457 | 0.00 | 0.00 | 0.00 |
| *Phleum phleoides* | homogeneous | non-mycorrhizal | 10 | 0.064 | 0.537 | -0.175 | 325.09 | 0.00 | 0.00 |
| *Phleum phleoides* | heterogeneous | mycorrhizal | 1 | 0.170 | 1.249 | -0.583 | 2298.72 | 0.00 | 0.00 |
| *Phleum phleoides* | heterogeneous | mycorrhizal | 2 | 0.250 | 1.221 | -0.061 | 17498.60 | 0.00 | 0.00 |
| *Phleum phleoides* | heterogeneous | mycorrhizal | 3 | 0.179 | 2.253 | -0.022 | 10636.59 | 0.00 | 844.79 |
| *Phleum phleoides* | heterogeneous | mycorrhizal | 4 | 0.323 | 1.393 | 0.408 | 10353.60 | 0.00 | 0.00 |
| *Phleum phleoides* | heterogeneous | mycorrhizal | 5 | 0.327 | 1.579 | 0.037 | 1233.75 | 0.00 | 0.00 |
| *Phleum phleoides* | heterogeneous | mycorrhizal | 6 | 0.158 | 0.931 | 0.191 | 83224.91 | 0.00 | 0.00 |
| *Phleum phleoides* | heterogeneous | mycorrhizal | 7 | 0.112 | 0.173 | 1.313 | 162894.95 | 14452.76 | 0.00 |
| *Phleum phleoides* | heterogeneous | mycorrhizal | 8 | 0.306 | 1.054 | 0.314 | 22269.11 | 7493.47 | 3842.78 |
| *Phleum phleoides* | heterogeneous | mycorrhizal | 9 | 0.111 | 0.463 | 0.148 | 302484.63 | 289738.04 | 2447.16 |
| *Phleum phleoides* | heterogeneous | mycorrhizal | 10 | 0.043 | 0.271 | 0.646 | 481.48 | 0.00 | 0.00 |
| *Phleum phleoides* | heterogeneous | non-mycorrhizal | 1 | 0.026 | 0.155 | -0.344 | 0.00 | 0.00 | 0.00 |
| *Phleum phleoides* | heterogeneous | non-mycorrhizal | 2 | 0.174 | 0.934 | -0.292 | 2071.92 | 0.00 | 0.00 |
| *Phleum phleoides* | heterogeneous | non-mycorrhizal | 3 | 0.095 | 0.387 | 0.770 | 81.40 | 0.00 | 0.00 |
| *Phleum phleoides* | heterogeneous | non-mycorrhizal | 5 | 0.008 | 0.158 | -0.559 | 458.70 | 0.00 | 0.00 |
| *Phleum phleoides* | heterogeneous | non-mycorrhizal | 6 | 0.236 | 1.294 | 0.125 | 169.05 | 0.00 | 0.00 |
| *Phleum phleoides* | heterogeneous | non-mycorrhizal | 7 | 0.180 | 1.047 | -0.576 | 136.71 | 0.00 | 0.00 |
| *Phleum phleoides* | heterogeneous | non-mycorrhizal | 8 | 0.098 | 0.432 | -0.567 | 1868.57 | 0.00 | 0.00 |
| *Phleum phleoides* | heterogeneous | non-mycorrhizal | 9 | 0.521 | 1.715 | 0.009 | 0.00 | 0.00 | 0.00 |
| *Phleum phleoides* | heterogeneous | non-mycorrhizal | 10 | 0.191 | 1.070 | 0.177 | 50.87 | 0.00 | 0.00 |
| *Potentilla recta* | homogeneous | mycorrhizal | 1 | 1.122 | 4.069 | -0.356 | 12725.23 | 0.00 | 0.00 |
| *Potentilla recta* | homogeneous | mycorrhizal | 2 | 1.725 | 7.435 | 0.004 | 4832.20 | 10694.91 | 3491.02 |
| *Potentilla recta* | homogeneous | mycorrhizal | 3 | 1.608 | 8.108 | -0.118 | 5549.50 | 0.00 | 0.00 |
| *Potentilla recta* | homogeneous | mycorrhizal | 4 | 0.132 | 0.636 | 0.874 | NA | NA | NA |
| *Potentilla recta* | homogeneous | mycorrhizal | 5 | 1.331 | 4.603 | -0.016 | 2151.89 | 29173.26 | 0.00 |
| *Potentilla recta* | homogeneous | mycorrhizal | 6 | 2.183 | 13.010 | -0.437 | 3666.40 | 5194.73 | 0.00 |
| *Potentilla recta* | homogeneous | mycorrhizal | 7 | 0.927 | 5.255 | -0.394 | 1509.86 | 49416.84 | 0.00 |
| *Potentilla recta* | homogeneous | mycorrhizal | 8 | 0.251 | 1.372 | -0.754 | 22296.67 | 0.00 | 0.00 |
| *Potentilla recta* | homogeneous | mycorrhizal | 9 | 1.635 | 10.004 | -0.279 | 1190.34 | 14142.24 | 0.00 |
| *Potentilla recta* | homogeneous | mycorrhizal | 10 | 1.327 | 5.927 | -0.117 | 19112.12 | 4730.82 | 0.00 |
| *Potentilla recta* | homogeneous | non-mycorrhizal | 1 | 1.307 | 6.966 | 0.153 | 69.75 | 0.00 | 0.00 |
| *Potentilla recta* | homogeneous | non-mycorrhizal | 2 | 1.353 | 4.619 | -0.083 | 181.57 | 0.00 | 0.00 |
| *Potentilla recta* | homogeneous | non-mycorrhizal | 3 | 1.500 | 7.565 | -0.091 | 55.74 | 0.00 | 0.00 |
| *Potentilla recta* | homogeneous | non-mycorrhizal | 4 | 2.180 | 7.614 | -0.107 | 0.00 | 0.00 | 0.00 |
| *Potentilla recta* | homogeneous | non-mycorrhizal | 5 | 1.621 | 7.756 | -0.004 | 471.70 | 0.00 | 0.00 |
| *Potentilla recta* | homogeneous | non-mycorrhizal | 6 | 0.866 | 5.141 | -0.385 | 15.89 | 0.00 | 0.00 |
| *Potentilla recta* | homogeneous | non-mycorrhizal | 7 | 1.300 | 5.678 | -0.031 | 97.49 | 0.00 | 0.00 |
| *Potentilla recta* | homogeneous | non-mycorrhizal | 8 | 1.376 | 5.801 | -0.105 | 84.91 | 0.00 | 0.00 |
| *Potentilla recta* | homogeneous | non-mycorrhizal | 9 | 1.720 | 9.180 | -0.027 | 192.51 | 0.00 | 663.09 |
| *Potentilla recta* | homogeneous | non-mycorrhizal | 10 | 1.259 | 5.409 | 0.198 | 1107.15 | 0.00 | 0.00 |
| *Potentilla recta* | heterogeneous | mycorrhizal | 1 | 1.346 | 6.658 | 0.431 | 21474.35 | 8756.05 | 1997.92 |
| *Potentilla recta* | heterogeneous | mycorrhizal | 2 | 2.028 | 8.494 | 0.699 | 20361.95 | 14854.62 | 0.00 |
| *Potentilla recta* | heterogeneous | mycorrhizal | 3 | 2.175 | 7.213 | 0.449 | 75820.51 | 67303.40 | 1662.05 |
| *Potentilla recta* | heterogeneous | mycorrhizal | 4 | 1.142 | 3.791 | 0.212 | 99893.36 | 176082.92 | 2004.92 |
| *Potentilla recta* | heterogeneous | mycorrhizal | 6 | 1.744 | 7.441 | 0.510 | 52560.54 | 17990.44 | 0.00 |
| *Potentilla recta* | heterogeneous | mycorrhizal | 7 | 1.273 | 6.099 | 0.114 | 28176.56 | 4845.21 | 0.00 |
| *Potentilla recta* | heterogeneous | mycorrhizal | 8 | 1.454 | 5.864 | 0.259 | 69137.55 | 30453.12 | 6695.87 |
| *Potentilla recta* | heterogeneous | mycorrhizal | 9 | 1.125 | 6.459 | 0.663 | 77057.35 | 38109.47 | 4062.85 |
| *Potentilla recta* | heterogeneous | mycorrhizal | 10 | 2.211 | 7.137 | 0.281 | 33338.52 | 6506.97 | 0.00 |
| *Potentilla recta* | heterogeneous | non-mycorrhizal | 1 | 1.470 | 7.348 | -0.252 | 2061.27 | 0.00 | 0.00 |
| *Potentilla recta* | heterogeneous | non-mycorrhizal | 2 | 1.472 | 6.186 | 0.385 | 149.26 | 0.00 | 0.00 |
| *Potentilla recta* | heterogeneous | non-mycorrhizal | 3 | 1.450 | 8.037 | -0.038 | 0.00 | 0.00 | 0.00 |
| *Potentilla recta* | heterogeneous | non-mycorrhizal | 4 | 1.909 | 7.189 | 0.412 | 99.18 | 0.00 | 0.00 |
| *Potentilla recta* | heterogeneous | non-mycorrhizal | 5 | 1.092 | 6.048 | 0.284 | 0.00 | 0.00 | 0.00 |
| *Potentilla recta* | heterogeneous | non-mycorrhizal | 6 | 1.971 | 6.192 | 0.274 | 130.78 | 0.00 | 0.00 |
| *Potentilla recta* | heterogeneous | non-mycorrhizal | 7 | 2.474 | 12.565 | 0.393 | 204.12 | 5252.22 | 0.00 |
| *Potentilla recta* | heterogeneous | non-mycorrhizal | 9 | 3.508 | 19.236 | 0.126 | 363.55 | 0.00 | 0.00 |
| *Potentilla recta* | heterogeneous | non-mycorrhizal | 10 | 1.336 | 6.016 | 0.417 | 928.74 | 7760.44 | 0.00 |
| *Ranunculus acris* | homogeneous | mycorrhizal | 1 | 0.477 | 2.721 | 0.187 | 179685.03 | 221418.93 | 0.00 |
| *Ranunculus acris* | homogeneous | mycorrhizal | 2 | 0.527 | 3.886 | 0.341 | 16346.59 | 0.00 | 0.00 |
| *Ranunculus acris* | homogeneous | mycorrhizal | 3 | 0.253 | 1.099 | -0.208 | 253013.93 | 95384.73 | 0.00 |
| *Ranunculus acris* | homogeneous | mycorrhizal | 4 | 0.295 | 1.555 | -0.096 | 18720.96 | 0.00 | 0.00 |
| *Ranunculus acris* | homogeneous | mycorrhizal | 5 | 0.064 | 0.309 | 0.206 | 118852.28 | 45321.08 | 0.00 |
| *Ranunculus acris* | homogeneous | mycorrhizal | 6 | 0.149 | 1.584 | -0.160 | 84935.04 | 0.00 | 0.00 |
| *Ranunculus acris* | homogeneous | mycorrhizal | 7 | 0.057 | 0.349 | -0.055 | 158059.75 | 50141.61 | 0.00 |
| *Ranunculus acris* | homogeneous | mycorrhizal | 9 | 0.213 | 1.058 | 0.076 | 23767.83 | 51808.91 | 0.00 |
| *Ranunculus acris* | homogeneous | mycorrhizal | 10 | 0.054 | 0.388 | -1.242 | 55419.91 | 22741.14 | 16022.07 |
| *Ranunculus acris* | homogeneous | non-mycorrhizal | 1 | 0.100 | 0.488 | -0.531 | 351.52 | 0.00 | 0.00 |
| *Ranunculus acris* | homogeneous | non-mycorrhizal | 3 | 0.192 | 1.784 | -1.032 | 9468.27 | 0.00 | 0.00 |
| *Ranunculus acris* | homogeneous | non-mycorrhizal | 4 | 0.040 | 0.241 | 1.996 | 100.26 | 174464.67 | 0.00 |
| *Ranunculus acris* | homogeneous | non-mycorrhizal | 7 | 0.070 | 0.301 | -0.758 | 83.35 | 0.00 | 0.00 |
| *Ranunculus acris* | homogeneous | non-mycorrhizal | 8 | 0.277 | 1.534 | -0.228 | 0.00 | 0.00 | 0.00 |
| *Ranunculus acris* | homogeneous | non-mycorrhizal | 9 | 0.110 | 1.027 | -0.210 | 0.00 | 0.00 | 0.00 |
| *Ranunculus acris* | homogeneous | non-mycorrhizal | 10 | 0.027 | 0.138 | -0.927 | 2123.58 | 0.00 | 0.00 |
| *Ranunculus acris* | heterogeneous | mycorrhizal | 1 | 0.423 | 2.850 | 0.120 | 86118.44 | 35060.58 | 9809.30 |
| *Ranunculus acris* | heterogeneous | mycorrhizal | 2 | 0.501 | 4.539 | -0.559 | 136477.45 | 70005.49 | 0.00 |
| *Ranunculus acris* | heterogeneous | mycorrhizal | 4 | 0.129 | 0.909 | 0.148 | 58076.44 | 138490.16 | 0.00 |
| *Ranunculus acris* | heterogeneous | mycorrhizal | 6 | 0.574 | 0.656 | 0.757 | 342626.60 | 157501.87 | 13948.84 |
| *Ranunculus acris* | heterogeneous | mycorrhizal | 7 | 1.272 | 11.493 | 0.574 | 12570.20 | 0.00 | 0.00 |
| *Ranunculus acris* | heterogeneous | mycorrhizal | 8 | 0.420 | 1.967 | 0.189 | 47790.79 | 39114.66 | 0.00 |
| *Ranunculus acris* | heterogeneous | mycorrhizal | 9 | 0.185 | 1.559 | 0.379 | 5509.19 | 0.00 | 0.00 |
| *Ranunculus acris* | heterogeneous | mycorrhizal | 10 | 0.128 | 0.691 | 1.373 | 629725.75 | 52583.40 | 15582.59 |
| *Ranunculus acris* | heterogeneous | non-mycorrhizal | 1 | 0.244 | 1.582 | -0.294 | 101.22 | 0.00 | 0.00 |
| *Ranunculus acris* | heterogeneous | non-mycorrhizal | 4 | 0.092 | 0.329 | -1.095 | 369.24 | 0.00 | 0.00 |
| *Ranunculus acris* | heterogeneous | non-mycorrhizal | 6 | 0.050 | 0.372 | 1.124 | 698.14 | 0.00 | 0.00 |
| *Ranunculus acris* | heterogeneous | non-mycorrhizal | 7 | 0.073 | 0.598 | 0.120 | 453.10 | 0.00 | 0.00 |
| *Ranunculus acris* | heterogeneous | non-mycorrhizal | 8 | 0.028 | 0.250 | -0.731 | 1702.78 | 0.00 | 0.00 |
| *Ranunculus acris* | heterogeneous | non-mycorrhizal | 9 | 0.143 | 0.570 | 0.262 | 0.00 | 0.00 | 0.00 |
| *Ranunculus acris* | heterogeneous | non-mycorrhizal | 10 | 0.090 | 0.622 | 0.642 | 17879.37 | 0.00 | 0.00 |

**Supplementary Table 4:** Number of pots included in the experiment and in the analyses. Here we refer to the linear analyses presented in Tables 2 and 3 and Table S5 in the Supporting Information. Thirty-seven plants died or were lost during the experiment, three samples were excluded due to missing root-associated AMF LSU copy number, and one sample was excluded due to very low DNA recovery during qPCR, resulting in a final sample size of 319 pots. Sample size differed slightly among analyses because of two additional missing biomass measurements (one shoot and one root measurement from the central pot).

| Analyses | Number of pots (n) |
| --- | --- |
| Initial sample size | 360 |
| Final sample size | 319 |
| Root and Shoot biomass analyses | 318 |
| Root:shoot ratio analyses | 317 |
| Root foraging precision analyses | 319 |

**Supplementary Table 5:** Linear model analyses of the effects of mycorrhizal root colonisation (Mycorrhiza: root-associated AMF LSU copy number of inoculated pots), plant species identity (Species) and nutrient treatment (Nutrient: homogeneous vs. heterogeneous) on the root and shoot biomass, root:shoot (R:S) ratio and root foraging precision of plants. For these analyses we used only data from AMF inoculated pots. Significant results are in bold. Root and shoot biomass and R:S ratio were log-transformed. The root-associated AMF LSU copy number was cube-root transformed.

|  |  | Df | Sum Sq | Mean Sq | F value | Pr(>F) |
| --- | --- | --- | --- | --- | --- | --- |
| Root biomass | **Mycorrhiza** | 1 | 8.43 | 8.43 | 14.39 | **<0.001** |
|  | **Species** | 8 | 153.47 | 19.18 | 32.73 | **<0.001** |
|  | **Nutrient** | 1 | 3.46 | 3.46 | 5.90 | **0.017** |
|  | Mycorrhiza * Species | 8 | 3.54 | 0.44 | 0.75 | 0.644 |
|  | Mycorrhiza * Nutrient | 1 | 0.07 | 0.07 | 0.11 | 0.736 |
|  | Species * Nutrient | 8 | 4.00 | 0.50 | 0.85 | 0.559 |
|  | Mycorrhiza * Species * Nutrient | 8 | 4.65 | 0.58 | 0.99 | 0.445 |
|  | Residuals | 124 | 72.68 | 0.59 |  |  |
| Shoot biomass | **Mycorrhiza** | 1 | 12.13 | 12.13 | 19.98 | **<0.001** |
|  | **Species** | 8 | 166.98 | 20.87 | 34.40 | **<0.001** |
|  | **Nutrient** | 1 | 2.69 | 2.69 | 4.44 | **0.037** |
|  | Mycorrhiza * Species | 8 | 6.72 | 0.84 | 1.39 | 0.210 |
|  | Mycorrhiza * Nutrient | 1 | 0.71 | 0.71 | 1.17 | 0.282 |
|  | Species * Nutrient | 8 | 6.80 | 0.85 | 1.40 | 0.203 |
|  | Mycorrhiza * Species * Nutrient | 8 | 5.65 | 0.71 | 1.16 | 0.327 |
|  | Residuals | 124 | 75.25 | 0.61 |  |  |
| R:S ratio | Mycorrhiza | 1 | 0.33 | 0.33 | 1.86 | 0.175 |
|  | **Species** | 8 | 27.56 | 3.45 | 19.18 | **<0.001** |
|  | Nutrient | 1 | 0.05 | 0.05 | 0.27 | 0.606 |
|  | Mycorrhiza * Species | 8 | 2.09 | 0.26 | 1.45 | 0.182 |
|  | Mycorrhiza * Nutrient | 1 | 0.34 | 0.34 | 1.89 | 0.172 |
|  | Species * Nutrient | 8 | 1.48 | 0.19 | 1.03 | 0.418 |
|  | Mycorrhiza * Species * Nutrient | 8 | 1.97 | 0.25 | 1.37 | 0.217 |
|  | Residuals | 124 | 22.28 | 0.18 |  |  |
| Root foraging precision | Mycorrhiza | 1 | 0.12 | 0.12 | 0.69 | 0.409 |
|  | Species | 8 | 0.96 | 0.12 | 0.71 | 0.682 |
|  | **Nutrient** | 1 | 3.71 | 3.71 | 22.07 | **<0.001** |
|  | Mycorrhiza * Species | 8 | 2.66 | 0.33 | 1.98 | 0.054 |
|  | Mycorrhiza * Nutrient | 1 | 0.02 | 0.02 | 0.12 | 0.728 |
|  | **Species * Nutrient** | 8 | 3.00 | 0.38 | 2.23 | **0.029** |
|  | Mycorrhiza * Species * Nutrient | 8 | 1.12 | 0.14 | 0.83 | 0.575 |
|  | Residuals | 124 | 20.84 | 0.17 |  |  |

**Supplementary Table 6.** Correlation coefficients for the relationships between root, shoot and whole plant biomass.

|  | Shoot biomass | Root biomass | Whole plant biomass |
| --- | --- | --- | --- |
| Shoot biomass | 1 | 0.817 | 0.997 |
| Root biomass | 0.817 | 1 | 0.859 |
| Whole plant biomass | 0.997 | 0.859 | 1 |

**Supplementary Table 7.** The percentage and number of pots with mycorrhizal fungal taxa detected using qPCR for each plant species and for all plant species; calculated for heterogeneous and homogeneous pots together and for the mycorrhizal treatment only.

|  | Colonised pots | | |
| --- | --- | --- | --- |
| Plant species  (number of pots) | *Funneliformis mosseae*  % of pots colonised (number of pots colonised) | *Rhizophagus irregularis*  % of pots colonised (number of pots colonised) | *Entrophospora claroidea*  % of pots colonised (number of pots colonised) |
| *Anthoxanthum odoratum* (19) | 100% (19) | 42% (8) | 21% (4) |
| *Centaurea jacea* (19) | 100% (19) | 74% (14) | 26% (5) |
| *Linaria vulgaris* (17) | 94% (17) | 50% (9) | 44% (8) |
| *Lotus corniculatus* (18) | 100% (18) | 61% (11) | 6% (1) |
| *Lythrum salicaria* (19) | 100% (19) | 5% (1) | 5% (1) |
| *Melilotus officinalis* (13) | 92% (12) | 31% (4) | 15% (2) |
| *Phleum phleoides* (19) | 95% (18) | 42% (8) | 32% (6) |
| *Potentilla recta* (18) | 100% (18) | 83% (15) | 33% (6) |
| *Ranunculus acris* (17) | 100% (17) | 71% (12) | 24% (4) |
| All species (159) | 98% (157) | 51% (82) | 23% (37) |

## 1.2 Supplementary Figures

**Supplementary Figure 1**. **A.** Small perforated pot used for planting seedlings to allow the establishment of mycorrhizal symbiosis with arbuscular mycorrhizal fungi over six weeks. Each pot was perforated on two opposite sides and inserted into a similar non-perforated pot to keep the substrate in darkness. **B.** Large 3-L pots in the centre of each of which is a small, perforated pot containing a plant. We installed drip irrigation on the opposite sides of each large pot so that the perforated sides of the small pot were directed towards the drippers. For a detailed description see the Methods section.

**Supplementary Figure 2.** The effect of nutrient treatment on root foraging precision of different plant species. Root foraging was generally higher in the heterogeneous treatment, but it differed between species. Red boxplots represent the heterogeneous nutrient treatment; blue boxplots represent the homogeneous nutrient treatment. We calculated root foraging precision in each pot as the decadic logarithm of N/W, where N was the biomass of dried roots from the nutrient-rich half of a given pot and W was the biomass of dried roots from the nutrient-poor half of the same pot. Values above zero indicate that plants developed more roots in the nutrient-rich half of the pot and values below zero indicate that plants developed more roots in the nutrient-poor half of the pot.

# 2 References

Koske, R.E., Gemma, J.N. (1989). A modified procedure for staining roots to detect VA mycorrhizas. Mycol. Res. 92, 486–488. https://doi.org/10.1016/S0953-7562(89)80195-9

McGonigle, T.P., Miller, M.H., Evans, D.G., Fairchild, G.L., Swan, J.A. (1990). A new method which gives an objective measure of colonization of roots by vesicular–arbuscular mycorrhizal fungi. New Phytol. 115, 495–501. https://doi.org/10.1111/j.1469-8137.1990.tb00476.x

Soudzilovskaia, N.A., Vaessen, S., Barcelo, M., He, J., Rahimlou, S., Abarenkov, K., Brundrett,	M.C., Gomes, S.I.F., Merckx, V., Tedersoo, L. (2020). FungalRoot: global online	database of plant mycorrhizal associations. New Phytol. 227, 955–966.	https://doi.org/10.1111/nph.16569

Thonar, C., Erb, A., and Jansa, J. (2012). Real-time PCR to quantify composition of arbuscular mycorrhizal fungal communities—marker design, verification, calibration and field validation. *Molecular Ecology Resources* 12, 219–232. doi: 10.1111/j.1755-0998.2011.03086.x

Weiser, M., Koubek, T., Herben, T. (2016). Root foraging performance and life-history traits.	Front. Plant Sci. 7:779. https://doi.org/10.3389/fpls.2016.00779
